# Identification of VHY/Dusp15 as a Regulator of Oligodendrocyte Differentiation through a Systematic Genomics Approach

**DOI:** 10.1371/journal.pone.0040457

**Published:** 2012-07-11

**Authors:** Fanny Schmidt, Monique van den Eijnden, Rosanna Pescini Gobert, Gabriela P. Saborio, Susanna Carboni, Chantal Alliod, Sandrine Pouly, Susan M. Staugaitis, Ranjan Dutta, Bruce Trapp, Rob Hooft van Huijsduijnen

**Affiliations:** 1 Merck Serono S.A., Geneva, Switzerland; 2 Department of Neurosciences, Lerner Research Institute, Cleveland Clinic, Cleveland, Ohio, United States of America; University of Massachusetts Medical, United States of America

## Abstract

Multiple sclerosis (MS) is a neuroinflammatory disease characterized by a progressive loss of myelin and a failure of oligodendrocyte (OL)-mediated remyelination, particularly in the progressive phases of the disease. An improved understanding of the signaling mechanisms that control differentiation of OL precursors may lead to the identification of new therapeutic targets for remyelination in MS. About 100 mammalian Protein Tyrosine Phosphatases (PTPs) are known, many of which are involved in signaling both in health and disease. We have undertaken a systematic genomic approach to evaluate PTP gene activity in multiple sclerosis autopsies and in related *in vivo* and *in vitro* models of the disease. This effort led to the identification of Dusp15/VHY, a PTP previously believed to be expressed only in testis, as being transcriptionally regulated during OL differentiation and in MS lesions. Subsequent RNA interference studies revealed that Dusp15/VHY is a key regulator of OL differentiation. Finally, we identified PDGFR-beta and SNX6 as novel and specific Dusp15 substrates, providing an indication as to how this PTP might exert control over OL differentiation.

## Introduction

**Figure 1:**
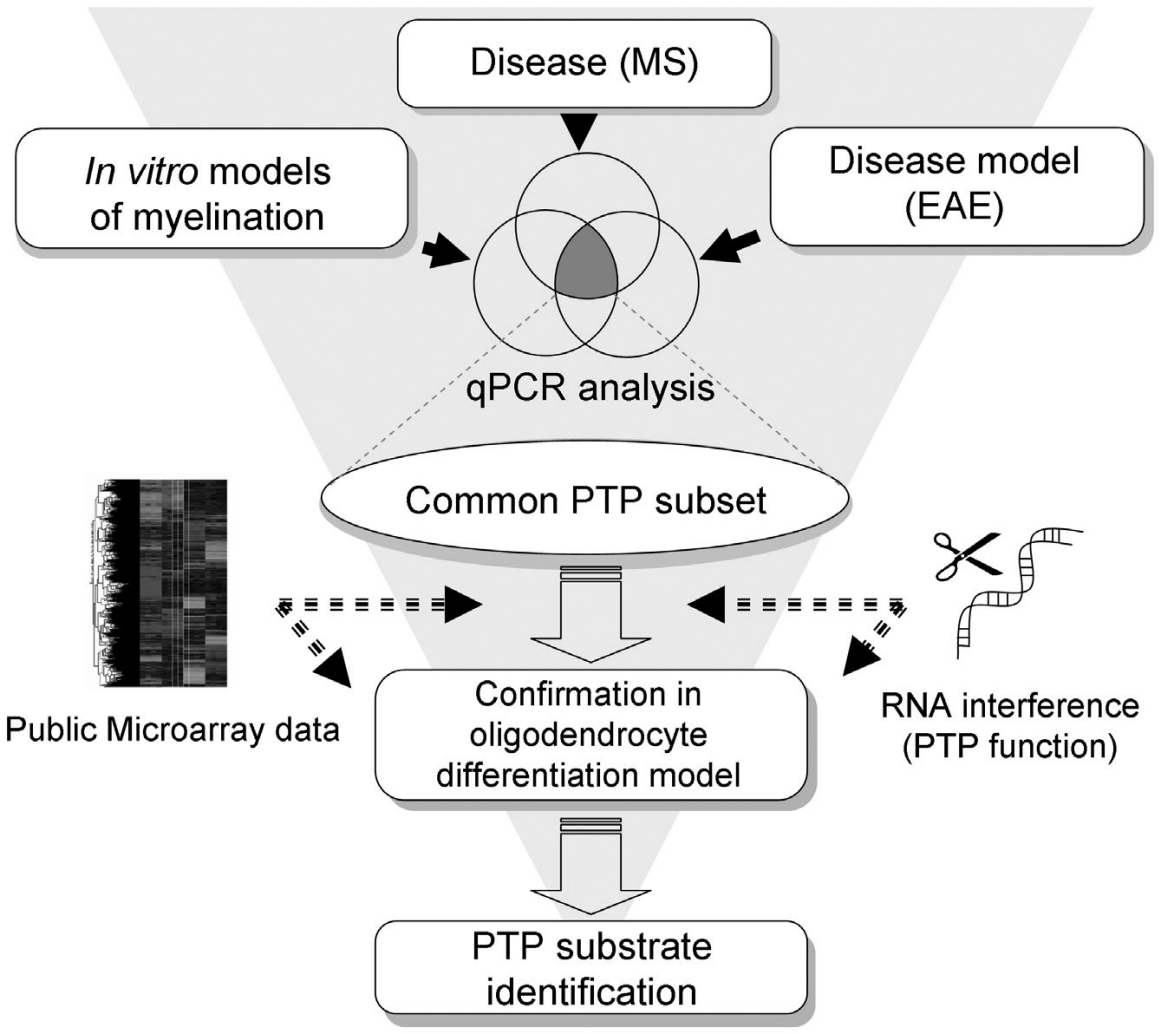
Schematic overview of the experimental approach.

Multiple Sclerosis (MS) is a chronic inflammatory, demyelinating and neurodegenerative disease characterized by immune system defects leading to axonal demyelination and neuronal degeneration. The early “relapsing-remitting” (RR-MS) stage of the disease has a clear autoimmune component that is adequately modeled in mice undergoing experimental allergic encephalitis (EAE) following immunization with myelin components. However, patients often shift to a secondary progressive (SP-MS) stage of the disease, where autoimmune involvement is less obvious, as evidenced by the lack of response to immunomodulatory treatments. SP-MS lesions often demonstrate a lack of remyelination, despite the recruitment of oligodendrocyte precursor cells (OPCs) in the peri-lesional area, as observed in post-mortem analyses [1], [2]. There is increasing evidence that OPC differentiation is under negative control [3], [4], [5]; repression that occurs in pathological conditions could be mediated by exogenous inhibitors like e.g myelin debris [6], semaphorin3A [7], or an accumulation of hyaluronan [8]. It is expected that agents that enhance OPC differentiation by overcoming these inhibitory factors would bring therapeutic benefit in MS, however this process is still poorly understood and useful drug targets need to be identified [9], [10], [11]. Several signaling cascades have been identified as drivers of oligodendrocyte (OL) differentiation and myelination that involve protein tyrosine kinases (PTKs) such as mitogen-associated protein kinases (MAPKs) [12], [13], [14], Phosphatidyl-Inositol-3-kinase (PI3K) [15], Protein kinases A/C (PKA/PKC) [16], [17], Focal Adhesion Kinase (FAK) [18] or Fyn kinase [19]. Optimal timing for the myelination process requires dynamic and adaptative systems involving interaction with other glial cells and axons to trigger the OL differentiation machinery. Among these processes, phosphorylation of tyrosine residues plays an essential role in numerous crucial cell signaling pathways regulated by the balanced activities of multiple PTKs and protein tyrosine phosphatases (PTPs), which are all members of two similarly-sized (∼100) gene families. Interest in signaling by PTPs has increased during the last decade, but is still focused on a small subset. Moreover, their roles in OLs are poorly understood [20], [21]. Genome-wide association studies (GWAS) have linked several PTPs to MS pathogenesis [22], [23], [24], [25] but the biological basis for these obervations remains often unclear. The importance of PTPs also emerges from microarray analyses that monitor OPC differentiation e.g. [26]. We therefore focused our study on the role of PTPs, which naturally counterbalance tyrosine phosphorylation cascades. Since OPCs may rely on and express different PTP subsets *in vitro* and *in vivo,* we focused our study on the PTP family with the aim of identifying OPC-differentiation regulators potentially relevant for the failure of OPCs differentiation in MS. Using transcriptional approaches, we investigated PTP expression profiles differences in MS lesions autopsies, during experimental autoimmune encephalomyelitis (EAE) in mice and during differentiation under non-inflammatory conditions in mouse embryonic mixed cortical cultures from newborn rats. The comparison of the three differential gene expression profiles obtained using q-PCR, led to the selection of a small PTP subset, whose functional involvement during *in vitro* OL differentiation was further assessed using a systematic RNA interference approach (see Figure 1 for an outline of the approach). VHY/Dusp15, a PTP previously only reported to be expressed in testis, unexpectedly emerged as the most promising functional candidate playing a master role in OL differentiation and undergoing expression modulation in MS. We finally identified potential Dusp15/VHY substrates that suggest an association with PDGFR-β and sorting nexin 6 (SNX6) signaling.

## Results

### A PTP Family-wide Expression Screen in MS-related Samples

While a considerable volume of microarray data is available, such arrays often do not cover all PTP genes. In addition, the limit of detection in this approach is lower than for other methods (Northern blotting or RT-PCR), while there is also a risk of detecting transcripts that do not encode functional proteins. With the aim of evaluating expression changes within the protein tyrosine phosphatase (PTP) family in MS-related samples, we therefore developed a semi-automated q-PCR method in a 384-well plate format allowing the determination of mRNA expression of 96 different genes including 92 human or mouse PTPs and 4 housekeeping genes (HKGs) in technical quadruplicates. The specificity of the entire set of oligonucleotides was validated by analysis of the cDNA fragment thermal dissociation curves while the distribution of oligonucleotide pairs was automated to minimize pipetting errors and distributed volume variation and to allow for robust quantification (data not shown). The entire oligonucleotide set including supplier references is available in Table S1.

The expression variation of PTP family members was first analyzed in 18 RNA samples extracted from white and gray matter tissues from control and MS autopsy brains. Both MS and control brain tissues were stained for myelin, and areas of demyelination (referred as lesions) were identified. Adjoining areas from MS brains with no loss of myelin was selected as “normal appearing tissue”. Identified regions were excised and total RNA was extracted from lesioned white/gray matter (*WML*/*GML*), from non-lesioned white/gray matter designated as normal appearing (*NAWM*/*NAGM*) and white/grey matter from control autopsies (*CWM*/*CGM*). Demographics of the control and MS patients are listed in Table 1.

**Table 1:** Clinical data of MS and control autopsies included in the study.

Patientno.	Age (years)	Sex	Samples*	Disease
1	52	F	*CWM*-1/ *CGM*-1	Myocardial infraction
2	53	F	*CWM*-2/ *CGM*-2	Cardiac thrombosis
3	77	M	*CWM*-3/ *CGM*-3	Mesenteric bleeding
4	46	M	*NAWM*-1/ *WML*-1	Secondary progressive MS
5	51	F	*NAWM*-2/ *WML*-2	Secondary progressive MS
6	63	F	*NAWM*-3/ *WML*-3	Secondary progressive MS
7	53	F	*NAGM*-1/*GML*-1	Secondary progressive MS
8	52	M	*NAGM*-2/*GML*-2	Secondary progressive MS
9	52	M	*NAGM*-3/*GML*-3	Secondary progressive MS

* *CWM*: Control white matter; *CGM*: Control gray matter; *NAWM*: Normal appearing white matter; *WML*: White matter lesioned; *NAGM*: Normal appearing gray matter; *GML*: Gray matter lesioned.

**Figure 2:**
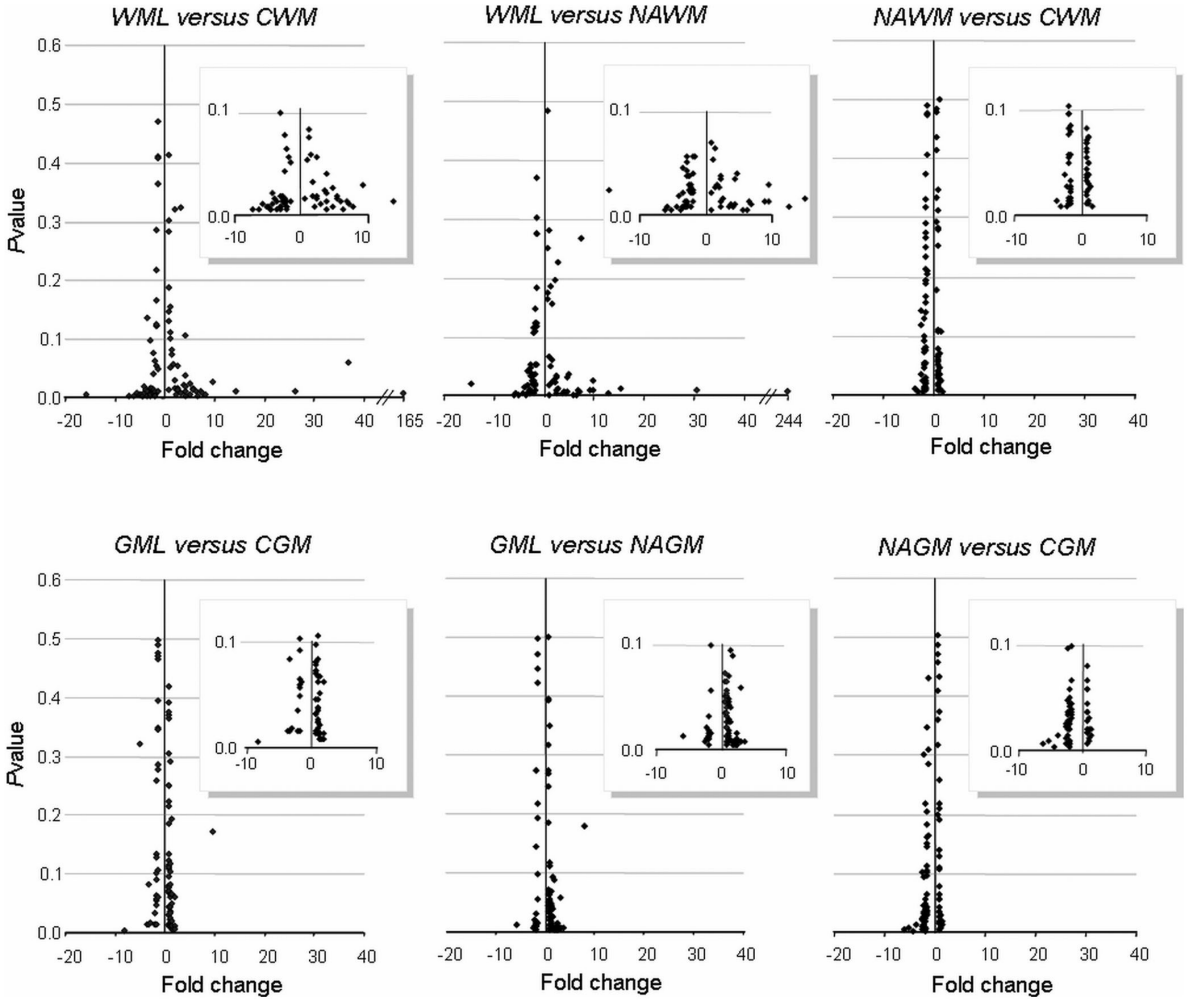
Differential gene expression of PTP family members in MS white and gray matter lesions. Data were expressed as a *ratio* of % expression *vs* HKGs between two samples (fold change) and represented in a volcano plot diagram (y: *P*value; x: fold change). An intensive modulation is characterized by spread values close to the x axis (magnification in square). *NAWM*, Normal-appearing White Matter; *NAGM*, Normal-appearing Grey Matter; *WML*, White Matter Lesion; *GML*, Gray Matter Lesion; *CGM*, Control gray Matter; *CWM*, Control White Matter. Major changes occur in lesioned areas and are more intensive in white matter.

**Figure 3:**
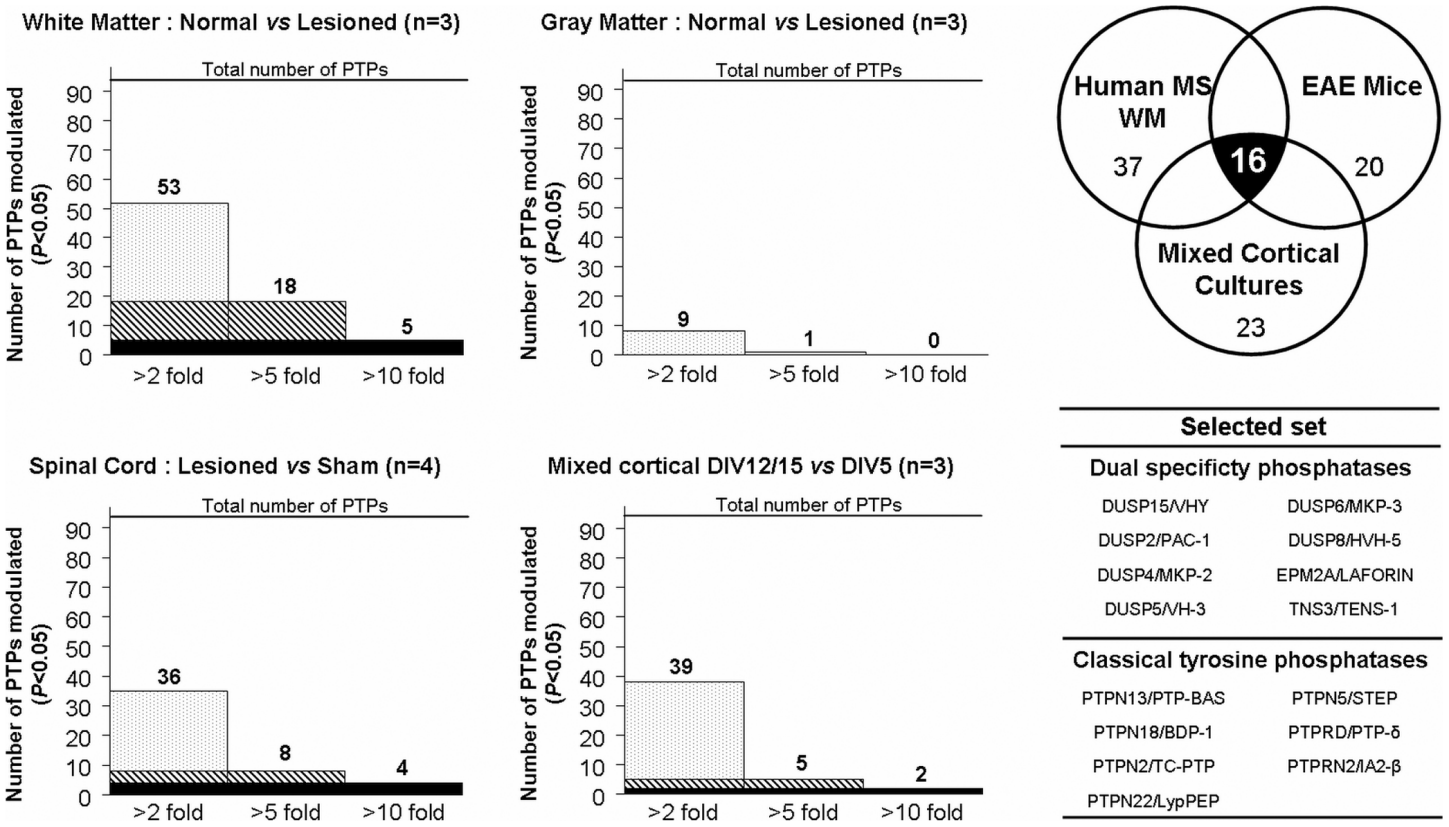
Comparative q-PCR-based differential gene expression and selection of PTPs of interest. The number of PTPs modulated are indicated for each sample type and classified by fold induction range. A gene is considered as significantly modulated when *P*val<0.05. Statistical analysis was performed using one tail student’s *t*-tests. A Venn diagram was used to represent the 16 PTPs modulated within the three sample types.

The mRNA expression profile of the 92 PTPs was determined in the 18 samples and results were calculated as fold changes in the *ratio* between the expression level in *WML* and either the expression level in the *NAWM* or the *CWM*. The process was repeated for gray matter tissues (Figure 2). The expression profile of the PTP family in MS *NAWM/NAGM* and in non-MS *CWM/CGM* were mostly identical, except for a few PTPs that were slightly modulated in gray matter (<5 fold change), suggesting that PTP expression is not dramatically modulated in absence of MS plaque formation in white and gray matter. Interestingly, expression of PTPs is extensively modulated in *WML* when compared to *CWM* and *NAWM.* In *WML*, 52 PTPs were found to be modulated at least two-fold, 18 PTPs were changed by >5-fold and five PTPs modulated by >10 fold (*P<*0.05) (Figures 2 and 3). This was in contrast to *GML*, where we observed only nine PTPs modulated by >2-fold and only one PTP modulated >5-fold (*P<*0.05) (Figures 2 and 3). These results provide evidence that major changes in expression levels of the PTP family members occur in MS *WML*. Among the most modulated (*P<*0.05) PTP genes were Ptpn18/BDP-1 (*WML vs CWM*/*NAWM* +14/+15 fold), Ptprq/PTP-GMC1 (−160/−243 fold), Ptpn22/LypPEP (−37/−78 fold), Dusp9/MKP-4 (−26/−30 fold), Ptprr/PTP-SL (−14/−9 fold), Dusp15/VHY (−8/−13 fold) and Ptpn5/STEP (−8/−5 fold). The complete dataset is available in Tables S2 (white) and S3 (gray matter).

**Figure 4:**
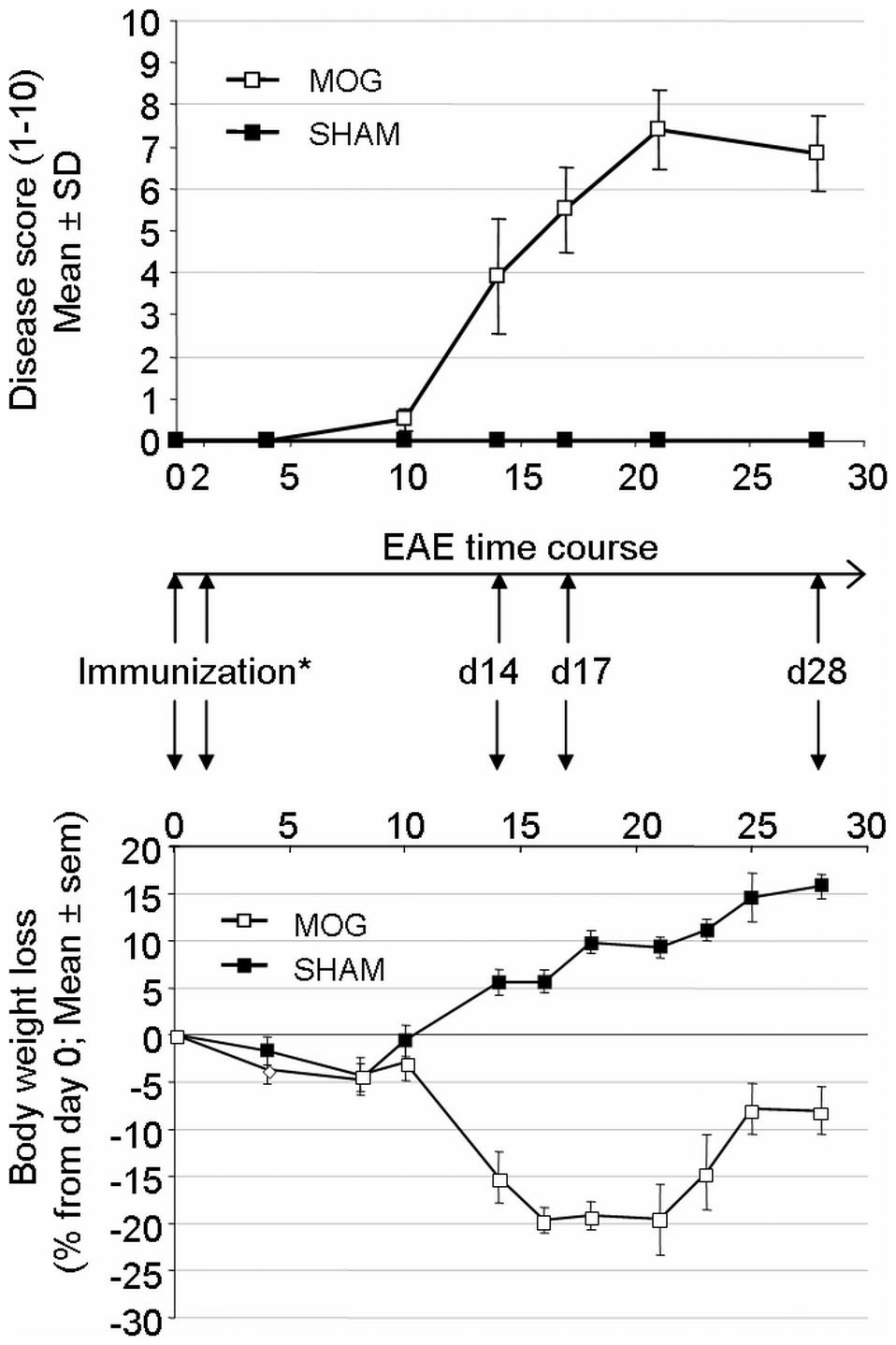
Disease course of mice undergoing EAE after immunization with rat MOG35-55. *Mice were immunized *s.c.* with rat MOG _35−55_. Pertussis toxin was administered *i.p.* at day 0 and day 2. 12 animals per group were included in this study. Neurological impairment was monitored using a ten-point standardized rating scale and expressed as Mean ± Standard Deviation. Spinal cord and cerebellum were taken at day14 (D14), day17 (D17) and day28 (D28) in MOG-induced (n = 4) and SHAM mice (n = 4). The body weight was monitored over the disease time course and the body weight loss ± SEM was reported. Disease onset occurred at day 10 and reached a maximum at day 22.

Differential gene expression analyses revealed extensive mRNA expression changes of PTP genes in *WML* of late-stage secondary progressive MS patients. In order to investigate how PTP gene expression changes during a time course of disease, we quantified PTP mRNA expression in CNS samples of C57/Bl6 mice undergoing a MOG-induced EAE, a well adopted model of demyelinating disease. Details of the model and sampling are shown in Figure 4. After EAE induction, mice were weighed daily and neurological impairment was quantified using a 10-points scale assessing paralysis (tail, hind and fore limbs), and death (Table 2). The study included 12 animals with EAE and 12 sham controls. Mice were euthanized and target tissues (spinal cord and cerebellum) were taken at three different time points (four animals per time point). mRNA expression analysis was performed using a 384-well format semi-automated q-PCR method which was ran using a validated mouse oligonucleotides set. Details for oligonucleotides are available in Table S1.

**Table 2:** Ten-point standardized rating scale for EAE clinical score monitoring.

	Score	Description
**Tail**	**0**	Tail erected
	**1**	Extremity of the tail flaccid with a tendency to fall
	**2**	Tail completely flaccid and drags on the table
**Hind limbs**	**0**	Energetic walk and doesn’t drag his paws
	**1**	Either one of the following test is positive: (i) flip test: a delay suggests hind-limb weakness, (ii) on the wire cage top: one or both limbs frequently slip between the bars, sign of partial paralysis
	**2**	Both previous tests are positive
	**3**	One or both hind limbs show signs of paralysis but some movement is preserved
	**4**	Both hind legs are paralyzed and the mouse drags them when moving
**Fore limbs**	**0**	Front paws active for grasping and walking and head erected
	**1**	Walking is difficult due to a weakness in one or both of the paws, another sign is head drooping
	**2**	One forelimb is paralyzed, head lost much of its muscle tone
	**3**	Cannot move, and food and water are unattainable
**Bladder**	**0**	Full control of bladder
	**1**	Mouse incontinent

The final score for each animal is determined by the addition of all the above mentioned categories. Note: Score  =  15 for dead animal.

**Figure 5:**
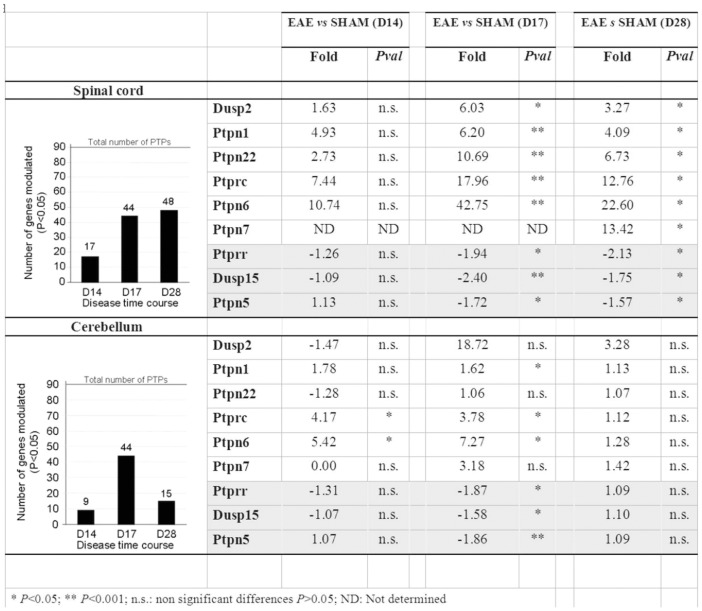
PTPs the most strongly modulated during EAE in mice spinal cord and cerebellum. Number of PTP genes significantly modulated during the EAE time course in the spinal cord and in the cerebellum has been monitored and represented in two graphics. The number of PTP genes modulated increases dramatically over time. At day 28, the number of PTP genes modulated decreases until a basal level in cerebellum but remains high in the spinal cord. The highest fold changes in gene expression versus Sham animals have been reported in the table. Most of these PTPs have already been described in inflammatpry processes. Statistical analysis were performed using student *t-*test.

The dataset shows that most PTP expression changes occur in spinal cord, with 35 PTP genes modulated (*P<*0.05) at least 2-fold, 18 genes modulated (*P<*0.05) at least 5-fold and four PTPs modulated (*P<*0.001) at least 10-fold. The six PTP genes that underwent the strongest modulation (>6 fold) in spinal cord during the time course of EAE were Ptpn22/LypPEP, Ptpn6/SHP-1, Ptpn7/LC-PTP, Ptprc/CD45, Ptpn1/PTP1B and Dusp2/PAC-1. The corresponding fold changes of these genes in spinal cord and cerebellum are reported in Figure 5 (See Table S4 for the complete dataset). All these PTPs have an immune cell-restricted expression pattern and have previously been described in inflammatory processes [27], [28]. These results thus likely reflect transient immune cells infiltration and microglia activation in the CNS during EAE [29], which has been documented as occurring predominantly in the spinal cords in this specific MOG-induced EAE model [30]. In order to also capture PTPs involved in remyelination/repair events we therefore also included modestly regulated PTPs in subsequent comparisons. We first compared the PTP gene sets obtained in MS and EAE samples, and 28 PTP genes were found to be commonly modulated (*P<*0.05). Three of the six PTP genes (Ptprr/PTP-SL, Dusp15/VHY and Ptpn5/STEP) that underwent the highest expression changes in MS white matter lesions were also found to be modulated in EAE mouse cerebellum and spinal cord (fold changes included in Table 3). It is interesting to note that PTPs known to be involved in the acute immune response like Ptprc/CD45, Ptpn22/LyPEP and Ptpn6/SHP1 are highly modulated at the time point of disease onset in EAE (14 days; Ptprc +7.4; Ptpn22+2.7; Ptpn6+10.7) unlike the modulation of Ptprr/PTP-SL, Dusp15/VHY and Ptpn5/STEP which occurs at a later time point (17 days and 28 days) during the peak of inflammation and beginning of recovery as described [31].

**Table 3:** Literature data for expression of selected PTPs in purified cells from rodent brain.

		Ptpn18	Ptprq	Ptpn22	Dusp9	Ptprr	Dusp15	Ptpn5
**In-house data (human)**	Fold change (*WML vs CWM*: *WML vs NAWM*)	−15/−14	+160/+243	+37/+78	+26/+30	+14/+9	+8/+13	+8/+5
**Cahoy ** ***et al*** **, 2007** a **(mouse) ** [**38**]	Astroglia (DIV12 culture, 69E)	40	No dataavailable	17	7	37	34	25
	Astroglia (DIV12 culture, 79 E)	68		16	19	34	15	26
	Astrocytes (P7; 56A)	23		13	39	38	207	261
	Astrocytes (P7; 81A)	18		16	36	41	183	272
	Astrocytes (P17; 72A)	35		31	32	34	206	177
	Neurons (P7; 81b)	38		8	28	498	147	1130
	Neurons (P16n; 61D)	22		12	15	489	206	1069
	OPCs (P17, 80O)	23		6	257	495	379	807
	OLs (P17, 80P)	26		9	759	432	3161	248
	Myelinating-OL (P17, 80Q)	59		11	76	172	3191	65
**Nielsen ** ***et al*** **,** **2006** b **(rat) (42)**	OPCs (A2B5^+^ O4-)	1.3	0.4	No data av.	0.5	1.2	0.4	1.3
	Myelinating OLs (A2B5+ O4^+^)	1.1	0.5		0.6	1.0	8.9	1.0

a Data extracted from GEO dataset GSE9566: GSM241928 (56A); GSM241929 (61D); GSM241930 (69E); GSM241931 (72A); GSM241932 (79E); GSM241933 (80O); GSM241934 (80P); GSM241935 (80Q); GSM241936 (81A); GSM241937 (81B). Samples designations correspond to the one used by the cited authors. Cell types were purified from post-natal mouse forebrains using FACS analysis. For more details see *Cahoy et al*, 2007.

b Data extracted from Geo dataset GDS2379: GSM138218-GSM138222 (n = 5); GSM138223-GSM138229 (n = 4). OL cell types were purified from post-natal P7 rats using FACS analysis. For more details see Nielsen *et al*, 2006.

**Figure 6:**
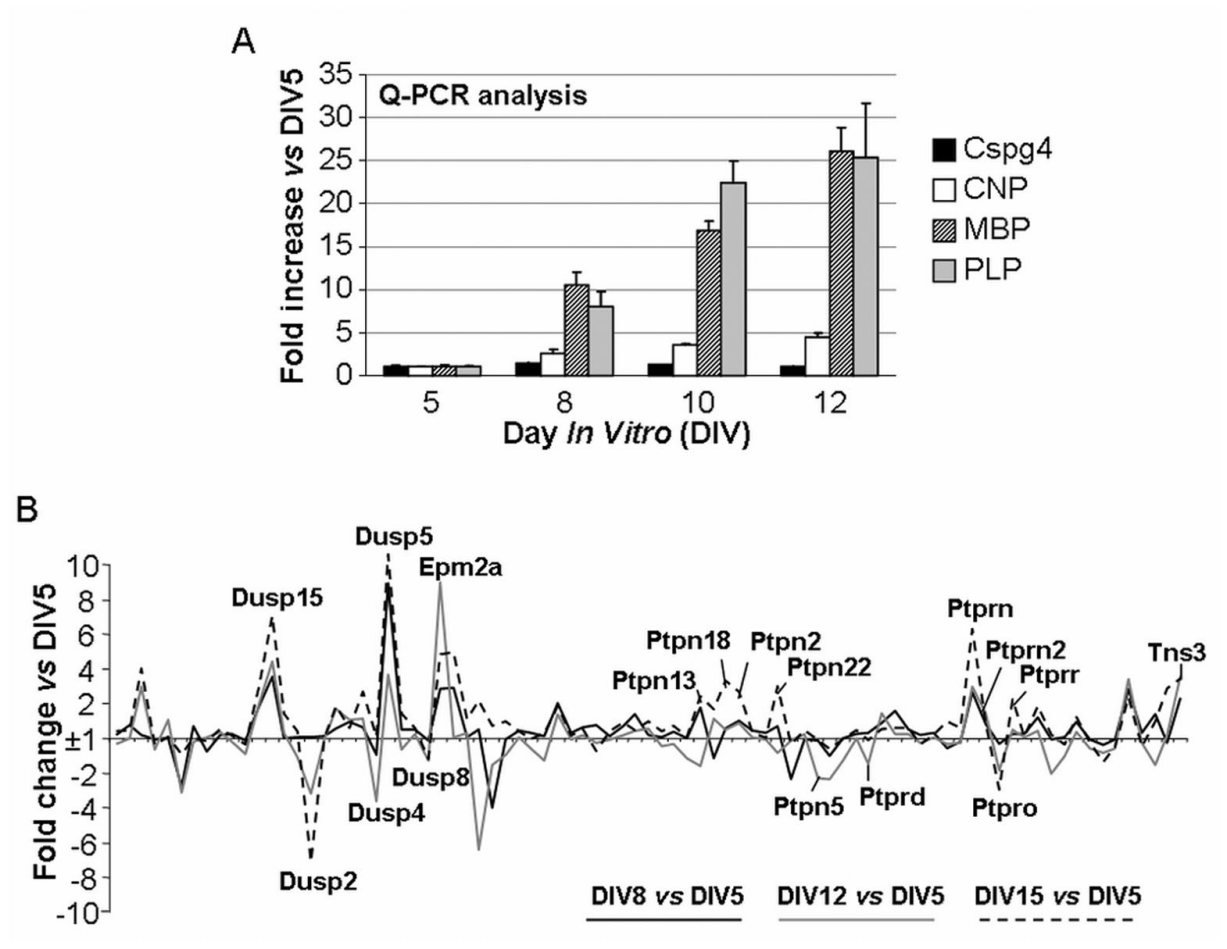
Expression of OL markers and PTP genes over time in fetal mouse mixed cortical cultures. **A**. mRNA expression of myelin markers CNP, MBP and PLP and the OPC marker cspg4 measured by qPCR. Results are expressed as fold change ± SEM corresponding to the *ratio* between % of gene expression *vs* HKGs at day *in vitro* (DIV) 8, 12 or 15 and % of gene expression *vs* HKGs at DIV5. Myelin markers expression increases during culture maturation, reflecting OPCs spontaneous differentiation over time. **B**. The modulation of the expression of the PTP gene family was monitored at DIV5, 8, 12 and 15 and expressed as fold increase *vs* DIV5. 39 PTPs were found to be modulated at least 2 fold in mouse mixed cortical culture. PTPs included in the final restrictive set are highlighted on the graphic.

In order to gain further insight into PTP gene expression in the myelination process under non-inflammatory conditions we then investigated their modulation over time in mouse embryonic mixed cortical cultures. As shown in Figure 6A, mRNAs for myelin-associated proteins CNP (2′, 3′-cyclic nucleotide 3′-phosphodiesterase), MBP (myelin basic protein) and PLP (proteolipid protein) undergo upregulation in this primary culture system over time reflecting OPC differentiation into mature OL. We found that the number of OPCs, as assessed by measurement of the expression of the OL progenitor marker NG2 (chondroitin sulphate proteoglycan, cspg4), remained stable over time suggesting a persistent compensatory proliferation of this cell subtype. The PTP family gene expression profile is shown in Figure 6B. Using the same data analysis procedure as for the two previously described q-PCR screenings, PTP genes were classified by fold changes (Figure 3). 38 PTP genes were found to be modulated by >2-fold and only five and two PTP genes were modulated by >5 and >10 fold, respectively, in these mouse mixed cortical cultures. Interestingly, we found that several PTP genes that were highly modulated in samples from MS patients were also modulated during normal OL differentiation, including, Dusp15/VHY, Dusp2/PAC-1 and Ptpn5/STEP (Figure 3). Two other PTPs appeared to be highly regulated in this system: Epm2a/LAFORIN which is expressed in different neural cell types, and Dusp5/hVH3, whose function is unknown. The entire dataset is available in Table S5.

A systemic analysis led us to further examine PTP genes which were found to be significantly modulated (*P<*0.05) in the three types of systems analyzed (MS patients, EAE mice and mixed cortical cultures) to identify PTPs potentially involved in OL differentiation.

From the 92 PTP gene set, aggregate data for 16 is reported in Table 4, Dusp15/VHY, Ptpn5/STEP and which notably includes Dusp2/PAC-1. In order to investigate the role of these PTPs in OL differentiation, their expression was measured during a time course of differentiation in the OL cell line Oli-neu, a validated model of *in vitro* OPC maturation [32], [33], [34], [35]. This OL cell line has been established by transfection of primary OPC-enriched cultures with the *t*-neu oncogene and is characterized by an overexpression of the ErbB2 receptor (EGFR family). Pharmacological inhibition of the ErbB2 receptor leads to Oli-neu differentiation. In our study, Oli-neu were allowed to differentiate using a treatment with the ErbB2 inhibitor PD174265 at 1 µM, and we measured (*i*) the basal level of the PTPs of interest in Oli-neu and (*ii*) their modulation during Oli-neu differentiation induced by PD174265.

**Table 4:** Fold-changes in mRNA expression for selected PTPs in several MS-related samples.

	MS patients *WML vs CTRL* WM		EAE mice spinal cord MOG *vs* SHAM		Mouse Mixed cortical DIVs *vs* DIV5		Oli-neu PD174265 *vs* CTRL	
	Fold increase	*Pval*	Fold increase	*Pval*	Fold increase	*Pval*	Fold increase	*Pval*
**DUSP4**	**6.13**	0.0110	**−1.56** ^ c^	0.0383	**−4.64** ^ b^	0.0157	**−5.88** ^ c^	0.0014
**DUSP6**	**5.88**	0.0142	**2.42** ^ b^	0.0028	**−1.62** ^ b^	0.0421	**−4.55** ^ c^	0.0010
**DUSP15**	**8.51**	0.0015	**−2.40** ^ b^	0.0005	**5.41** ^ b^	0.0151	**2.6** ^ c^	0.0327
**TNS3**	**1.72**	0.0281	**3.37** ^ b^	0.0002	**4.60** ^ b^	0.0061	**4.3** ^ b^	0.0129
**PTPN2**	**4.57**	0.0071	**1.74** ^ b^	0.0137	**1.99** ^ a^	0.0412	**−3.80** ^ a^	0.0104
**PTPN5**	**8.00**	0.0051	**−1.72** ^ b^	0.0050	**−3.29** ^ b^	0.0097	**−3.83** ^ c^	0.0219
**PTPRD**	**−4.12**	0.0006	**−2.44** ^ b^	0.0010	**−2.43** ^ b^	0.0129	**−3.39** ^ a^	0.0112
**PTPN13**	**−3.55**	0.0114	**−1.82** ^ c^	0.0026	**2.75** ^ a^	0.0272	**2.8** ^ b^	0.0224
**PTPN18**	**−15.47**	0.0038	**4.42** ^ a^	0.0225	**1.55** ^ a^	0.0105	^d^	n.s
**PTPRN2**	**7.09**	0.0012	**−2.24** ^ b^	0.0003	**2.46** ^ b^	0.0434	^e^	
**PTPN22**	**37.12**	0.0267	**10.69** ^ b^	0.0012	**1.67** ^ a^	0.0035	^d^	n.s
**DUSP5**	**−3.69**	0.0160	**3.22** ^ b^	0.0042	**11.28** ^ c^	0.0334	**−1.3** ^ c^	0.0400
**DUSP2**	**3.07**	0.0155	**6.03** ^ b^	0.0046	**−8.45** ^ c^	0.0025	^a^	n.s
**DUSP8**	**4.47**	0.0166	**−1.79** ^ c^	0.0005	**−2.50** ^ c^	0.0181	^d^	n.s
**EPM2A**	**3.08**	0.0114	**−1.93** ^ c^	0.0001	**10.01** ^ b^	0.0150	^d^	n.s
**PTPRR**	**14.62**	0.0089	**−1.94^ b^**	0.0059	**2.91^ c^**	0.0358	**−2.8** ^a^	0.0085
	*P*<0.05		*P*<0.05		*P*<0.05		*P*<0.05	
Additional PTP								
**PTPRO**	**1.10**	0.280	**1.45** ^ a^	0.0277	**−4.27** ^ c^	0.0135	**−1.6** ^ c^	0.0655
			^a^ 14 days post-immunization		^a^ DIV8/DIV5		^a^ 24 h post-treatment	
			^b^ 17 days post-immunization		^b^ DIV12/DIV5		^b^ 48 h post-treatment	
			^c^ 28 days post-immunization		^c^ DIV15/DIV5		^c^ 72 h post-treatment	
							^d^ no modulation at any time	
							^e^ not expressed	

Among the 16 PTPs extracted, one was not detected (Ptprn2/IA2-beta), six were detected at a very low level (Ptpn2/TC-PTP, Ptpn5/STEP, Ptprd/PTP-delta, Ptpn22/LypPEP, Ptprr/PTP-SL) and the nine other mRNAs were found to be moderately expressed, the two highest one being dusp6/MKP-3 and Tns3/TENS1. We then considered all PTPs detectable and followed their expression over the time course of differentiation in Oli-neu. Nine PTPs were found to be modulated during Oli-neu differentiation with fold changes comprised between 1.3 and 5.9 (*P<*0.05). Dusp5/hVH3 was removed from the set given its low modulation rate. Results are indicated in Table 4. Given the recent literature describing Ptpro/GLEPP-1 as a regulator of WnT signalling, microarray data from public databases, and previously published studies led us to include Ptpro/GLEPP-1 into the selected set of interest [36], [37].

### RNA Interference Screening

**Figure 7:**
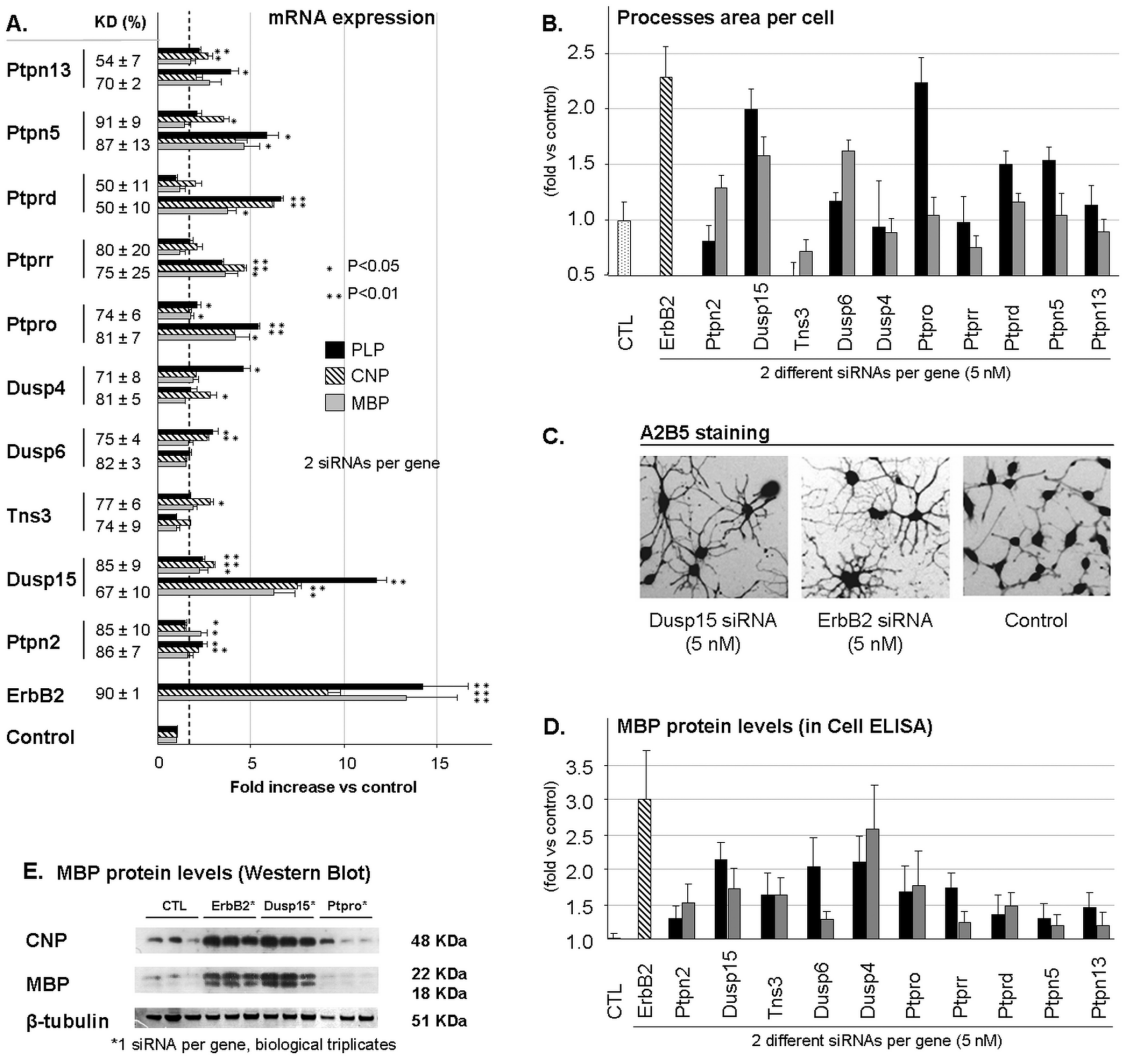
Effect of targeted silencing of 10 PTPs of interest on Oli-neu differentiation. A. mRNA expression analysis of three myelin markers MBP (myelin basic protein), CNP (2’, 3’-cyclic nucleotide 3’-phosphodiesterase), PLP (Proteolipid protein) in si-RNA-transfected olineu after 72 h. % of expression were calculated using the delta Ct method and expressed in fold change *versus* negative control as Mean ± SEM of three independent experiments performed in triplicate. Knock-down % (KD%) was calculated using the formula 100−(100 x Expression *vs* HKGs in sample/Expression *vs* HKGs in control) and was expressed as Mean ± SEM of the same three independent experiments. Statistical analysis was performed using one tail student *t*-tests. siRNAs targeting Dusp15/VHY induce myelin markers expression. B. Morphological analysis performed by using the Cellomics method at 72 h after transfection with the si-RNA. Processes area per cell defines the mean area regrouping processes surrounding the OL cell body. Each data point was obtained by analysis of at least 100 cells per well automatically and normalized by the number of cells. Results were normalized to negative controls and expressed as Mean ± SEM of at least three different experiments performed in quadruplicate. C. Images of Oli-neu non transfected (right) and transfected with ErbB2 si-RNA (center) or Dusp15/VHY si-RNA (left) immunostained against A2B5 with a Cy3-coupled (red) secondary antibody. Images were acquired using an inverted fluorescence microscope then the black and white mode was inverted using image analysis software to allow better processes visualization. Dusp15/VHY silencing induces Oli-neu morphological differentiation. D. In-Cell ELISA was performed on Oli-neu immunostained against MBP and revealed using the ABTS (2,2′-azino-bis-(3-ethylbenzothiazolin-6-sulfonic acid) colorimetric transformation by horseradish peroxidase coupled to a secondary antibody. Results were normalized to negative controls and expressed as Mean ± SEM of at least three different experiments performed in quadruplicate. E. Western blot analysis of MBP and CNP. Protein expression levels were compared to the internal control β-tubuline. Dusp15/VHY gene knockdown induces an increase in MBP and CNP protein levels.

Subsequently we assessed functional involvement of our ten-PTP genes set in OL differentiation using an RNA interference strategy on the Oli-neu cell line. Four si-RNAs directed against each gene were tested for their ability to knock down the mRNA of their target using real time quantitative PCR analysis and the two si-RNAs leading to the best percentage of knockdown (KD%) were selected for further analysis (data not shown)… KD% was calculated using the formula 100−(100 x Expression *vs* HKGs in sample/Expression *vs* HKGs in control) and expressed as mean ± SEM from three independent experiments. KD% values are reported in Figure 7A. To evaluate Oli-neu differentiation a first quantitative morphological analysis was performed allowing the quantification of OL processes elongation. This fully automated method (Cellomics® platform) uses fluorescence microscopy coupled with image analysis software. Oli-neu were cultured for 72 hours after transfection with si-RNAs then cells were fixed and immunostained for polysialogangliosidase A2B5 before morphological analysis. A2B5 is detectable both at the proliferating and differentiating states following treatment with Erbb2 inhibitor for 72 hours. An si-RNA directed against the ErbB2 receptor served as positive control for its ability to drive Oli-neu morphological and biochemical differentiation, characterized by OL processes elongation and myelin markers expression. All values were normalized to negative control corresponding to cultures transfected either with an si-RNA targeting the gene encoding luciferase or a mixture containing only transfection reagents (MOCK). No si-RNAs which were selected and tested were toxic for the cells at the concentration used. We observed that the silencing of some PTP genes was accompanied with outgrowth of processes. This effect was shown for Dusp15/VHY, Dusp6/MKP-3 and Ptprd/PTP-delta and supported by the similar effect induced by the two si-RNAs used. For other PTPs, some changes were observed with divergences between the two si-RNAs (Ptpro/GLEPP-1) leading to interpretation difficulties. These results are presented in Figure 7B. To complement these morphological observations, myelin markers MBP, CNP and PLP were quantified in knocked-down cultures using quantitative real-time PCR. From the ten PTPs silenced, only Dusp15/VHY knock-down was consistently confirmed to drive a differentiation process (Figure 7A). Figure 7C illustrates the morphological changes in native Oli-neu undergoing normal proliferation state (right), or after ErbB2 (center) or Dusp15/VHY (left) down-regulation. We found a good correlation between the expression of myelin markers with the morphological analysis for a corresponding si-RNA (Figure 7A read bottom to top *versus* Figure 7B read left to right). To also measure marker protein expression, MBP levels were first quantified using an *in-cell* ELISA on Oli-neu transfected with the same 20 different si-RNAs targeting the ten-PTP gene set. Good correlation between MBP protein levels measured by ELISA and mRNA expression was observed in Oli-neu where Dusp15/VHY, Ptpro/GLEPP-1 or Dusp4/MKP-2 genes were silenced (Figure 7D). On the other hand, differences between experiments were observed for some other PTP genes such as Ptpn5/STEP or Ptprd/PTP-delta. Throughout the different methods, only the results relative to Dusp15/VHY were consistent and were additionally confirmed by Western blot analysis showing a dramatic increase in MBP and CNP protein levels in si-RNA-transfected cells (Figure 7E). These results suggest a role of Dusp15/VHY in negative regulation of OL differentiation.

### Dusp15/VHY Expression during Oligodendrocyte Differentiation

**Figure 8:**
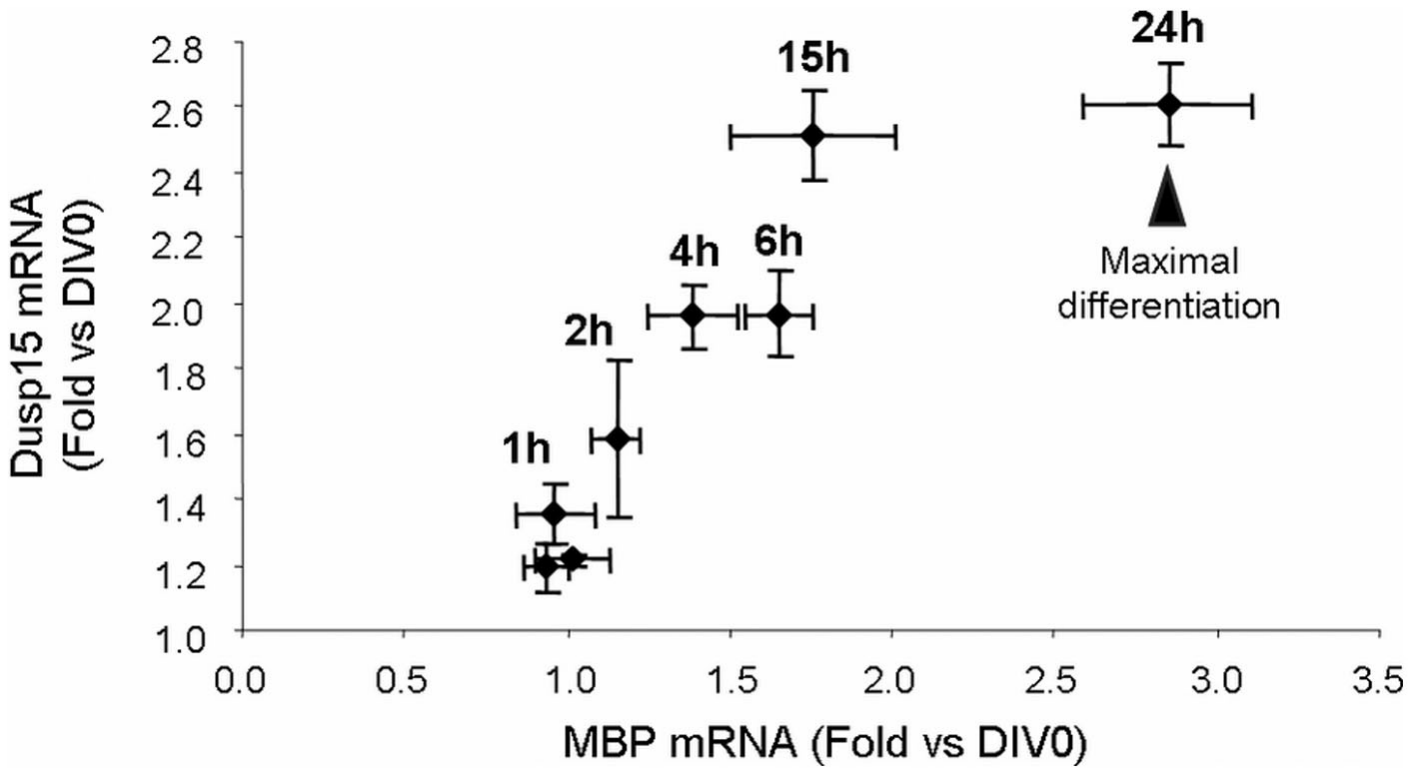
Correlation chart between MBP expression and dusp15 expression over a time course of differentiation in olineu. Values expressed as fold induction *versus* undifferentiated controls (starting cultures) and correspond to the Mean ± SD of two different experiments (n = 2). Dusp15/VHY expression increases with time and correlates with MBP expression during the first steps of Oli-neu differentiation then Dusp15 expression reaches a maximum at 15 h prior to the MBP expression peak occurring at 24 h.

**Figure 9:**
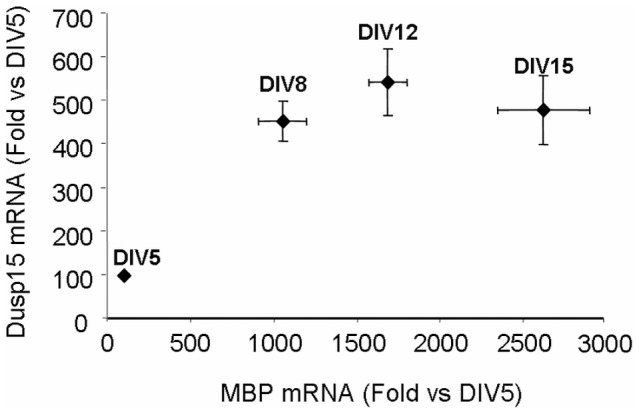
Correlation chart between MBP expression and dusp15 expression over a time course of differentiation in mouse primary cortical cultures. Values expressed as fold induction *versus* DIV5 cultures and correspond to the Mean ± SD of two different experiments (n = 3). Dusp15/VHY expression increases with time and correlates with MBP expression until DIV12 then reaches a maximum whereas MBP expression still increases until DIV15.

In order to investigate modulation of Dusp15/VHY expression during OL differentiation, quantitative RT-PCR was performed on Oli-neu cells undergoing differentiation after treatment with an ErbB2 inhibitor. Dusp15/VHY RNA transcription increased during Oli-neu-differentiation and correlated with an increase in MBP mRNAs levels, stabilizing when full differentiation was reached (Figure 8). This increase in Dusp15/VHY during OL differentiation is in good agreement with microarray data obtained from FACS-purified mouse OPCs, pre-OLs and myelinating OLs from post-natal brains [38] and from FACS-purified rat OPCs [39], as summarized in Table 3. An increase in Dusp15/VHY expression was also seen during a time course in mixed cortical cultures (Figure 9).

The link between myelin production and Dusp15/VHY expression also emerges from re-analysis of our recent microarray study where we used different pharmacological compounds to drive Oli-neu differentiation (19). The more extensive data presented in the present study, as summarized in Table 5, demonstrated a clear relationship between Dusp15/VHY expression and myelin production [26], [34].

**Table 5:** VHY/Dusp15 expression during Olineu differentiation.

			Treatment time course						MBP production at 72 h
			10 h		24 h		72 h		
**Pescini Gobert ** ***et al*** **, 2009^a^** **Joubert ** ***et al*** **, 2010^b^** **(Olineu)** [**26]**, [34****]		Insuline	−1.30		−1.30		−1.16		–
		Retinoic acid	1.03		1.13		1.39		–
		Forskolin	−1.48		2.02	*	2.09	**	+/−
		Dexamethasone	1.66		3.76	**	3.27	**	+++
		ErbB2 inhibitor (PD174265)	1.56		3.47	**	2.43	**	+++

a,b Data extracted from in-house microarray analysis of transcriptional changes in olineu differentiation induced with different classes of chemical inducers. MBP production is qualitatively reported and reflects the original quantitative data obtained by western blot analysis.

* *P*<0.05;

** *P*<0.001.

For more details please refer to the original publications ^a, b^.

We found that DUSP15 is upregulated in MS lesions, but was repressed in the EAE model (Table 4). This finding seems counterintuitive, however one should bear in mind that we are comparing an acute mouse disease model with advanced human disease. One critical difference is that the mouse model is acute: there is strong immune involvement, however myelin repair is almost complete. By contrast, human (postmortem) tissue is from late-stage patients that is associated with less involvement of the immune system and (but) failing remyelination. One explanation for the discrepancy therefore is that the cellular environment in the human disease (myelin debris? innate/microglia immune responses?) somehow stimulates DUSP15 expression, keeping oligodendrocytes in the undifferentiated state. The result may also simply reflect the different stages of oligodendrocyte differentiation in the two situations, low in (differentiated) mouse oligodendrocytes but high in human, stalled oligodendrocytes. Thus, DUSP15 may behave as GPR17, a cell-intrinsic timer of myelination [40].

### Dusp15/VHY Substrate Identification by Phospho-peptides Arrays

**Figure 10:**
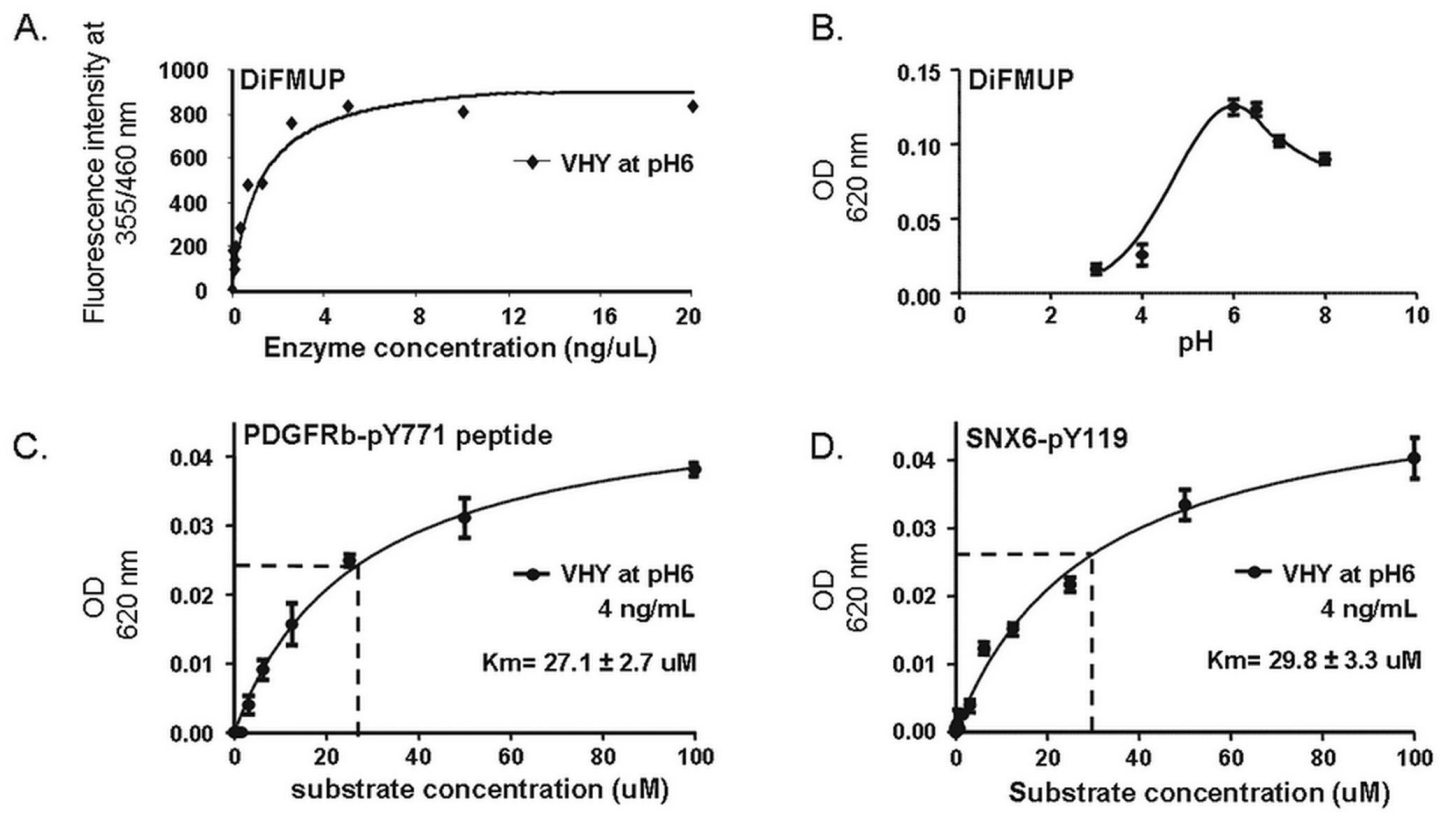
Phosphatase activity of GST-tagged full length Dusp15/VHY. A. Dusp15 phosphatase activity was assessed using the DiFMUP (6,8-difluoro-4-methyumbelliferyl phosphate) assay at the experimentally optimal enzyme concentration of 4 ng/mL. B. Optimal pH activity (pH 6) was determined by testing a pH range from pH 3 to 8. C. and D. Activity of Dusp15/VHY on phospho-peptides substrates corresponding respectively to pY_119_ and pY_771_ dephosphorylation sites of SNX6 (NED(pY_119_)AGYIIPPAP) and PDGFR-β (IESSN(pY_771_)MAPYD). VHY/Dusp15 was used at 4 ng/mL at pH6 and activity was detected using the Malachite Green phosphate detection assay. OD, Optical Density at 620 nm. Dissociation constant (Km) was calculated as the substrate concentration needed to reach V_max_/2 and expressed as Mean ± S_EM_ of three different experiments.

The functional role of Dusp15/VHY in cell signalling, whether in Ols or any other tissue is currently unknown. In order to understand which phosphorylation events are regulated by this phosphatase, we assayed a set of phospho-peptides as substrates for GST (Glutathione S-transferase)-tagged full length recombinant Dusp15 phosphatase. Recombinant Dusp15/VHY (AbNova) was obtained, tested for purity and identity by Western blot (not shown), and activity was preliminary tested in a DiFMUP (6,8-difluoro-4-methylumbelliferyl phosphate) *in vitro* dephosphorylation assay (Figure 10A, B). Enzymatic activity of Dusp15/VHY was previously described in a *p*NPP (*para*-nitrophenyl phosphate) dephosphorylation assay [41]. We found the optimal DiFMUP dephosphorylation was at pH 6 (Figure 10B), which correlates with literature data [41]. Optimal activity was determined at an enzyme concentration of 4 ng/µL. An intermediary concentration of 2 ng/µL was used in a 384-well format assay (Figure 10A).

**Figure 11:**
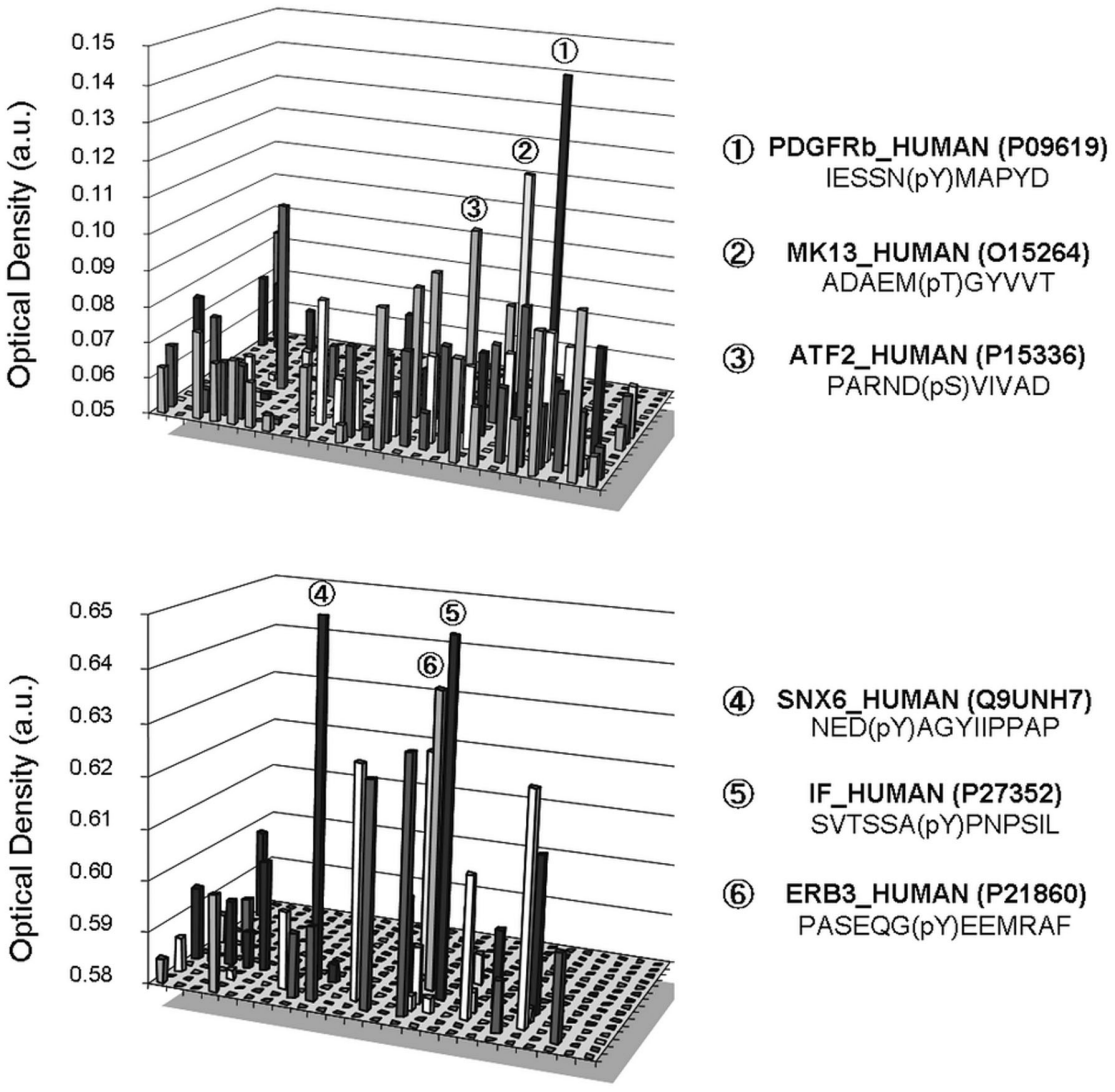
Identification of VHY potential substrates using phospho-peptides arrays. Each graphic correspond to the results expressed as optical density in arbitrary unites (a.u.) arising from two different arrays representing 720 different phospho-peptides. An arbitrary threshold allowed for the selection of the three phospho-peptides hits of each array namely (1) PDGFR-β (Platelet-derived Growth Factor Receptor beta); MK13 (MAPKinase 13/p38MAPKdelta); ATF2 (Activating Transcription Factor 2); SNX6 (Sorting Nexin 6); IF (Intrinsic factor); ErbB3 (v-erb-b2 erythroblastic leukemia viral oncogene homolog 3). Arrays were ran at pH6 with an enzyme concentration of 2 ng/mL.

The 384-well format phospho-peptide test plates were purchased from JPT Peptides Technologies. These contained 720 phospho-peptides that carry phospho-tyrosine (pY) or phospho-Ser/Thr (pS/pT) residues, corresponding to known, biologically relevant substrates. The phosphopeptides were screened at 0.5 µM on recombinant Dusp15/VHY. Phosphate release was quantified using the Malachite Green phosphate detection assay by measurement of the optical density at 620 nm after 24 hours incubation at room temperature. Dephosphorylation results in an increase in absorbance at 620 nm. We found several potential substrates including PDGFR-β (Platelet-derived Growth Factor Receptor type B), MK13 (p38MAPK-delta), ATF2 (Activating Transcription Factor 2), SNX6 (Sorting Nexin 6), IF (Intrinsic factor) and ErbB3 (v-erb-b2 erythroblastic leukemia viral oncogene homolog 3; Figure 11) with confirmation and Km determination for PDGFR and SNX6 as shown in Figure 10. As dual specificity phosphatases are generally known to be involved in the MAPK (Mitogen-activated Protein Kinase) signalling, it is not surprising to detect an activity on substrates like MK13 and ATF2. However, PDGFR-β and SNX6 are interesting substrates since the roles of these two proteins have not been studied so far in OLs.

To validate our substrates identification, peptides corresponding to PDGFR-β (pTyr771) and SNX6 (pTyr119) were dephosphorylated *in vitro* using the same GST-tagged full length Dusp15/VHY in a substrate dose response study. This allowed the confirmation of VHY/Dusp15 activity on these specific substrates and the determination of a Km of 27.1±2.7 µM for PDGFR-β Y771 and 29.8±3.3 µM for SNX6 Y119 (Figure 10C, D).

## Discussion

Several lines of evidence support the observation that remyelination in MS is partially impaired because of the inability of OPCs to differentiate into myelinating OLs [9], [10], [11], [3], [4], [5], [6], [8]. During the last decade, several inhibitory pathways of OL differentiation were identified, like Wnt [42], Notch-1 [43], [44] LINGO/RhoA [45] and others [46]. Considering that SNPs within the PTP family genes have been linked to MS pathogenesis and that several PTPs have been demonstrated already to be involved in OL differentiation, and also to discover relevant phosphorylation activities we focused our study on the PTP family with the aim to further identify OPC-differentiation regulators potentially relevant for the failure of OPCs differentiation in MS. VHY/Dusp15 has emerged as the most promising candidate.

### VHY/Dusp15 as Regulator of OL Differentiation

There are several other genes that are upregulated during oligodendrocyte differentiation (but) whose repression results in spontaneous differentiation. Two of these (MKP5, CNP) were previously described by us [26]; another example is GPR17 [40]. This evidence suggests, as noted earlier [26], that differentiation in oligodendrocytes is associated with the induction of a number of negative feedback loops (especially an increase in phospatase activity, likely associated with enhanced kinase activity).

Another PTP, namely PTPRZ was reported to be involved in oligodendrocyte proliferation and differentiation [47], [48], however we found no evidence for significant transcriptional modulation for this PTP in our models.

### Rationale for PDGR-β Signalling in OL Differentiation

Previously, Dusp15 expression had been demonstrated in testis only, whereas other organs failed to yield detectable Northern signals. Many genes are expressed in testis without obvious significance, and our work highlights the importance for surveying larger sets of cell types using more sensitive approaches for such genes. Two substrates of particular interest were found to be dephosphorylated by Dusp15/VHY, namely PDGFR-β and SNX6. However, the question whether Dusp15/VHY role in OLs differentiation is mediated through dephosphorylation of PDGFR-β and/or SNX6 (or yet undiscovered substrates) remains to be clarified. PDGF receptors (PDGFRs) have been extensively studied, especially in cancer, because of their role in cellular proliferation [49]. Study of PDGFRs in OLs has so far focused on the alpha than the beta isoform, since it is significantly more expressed in OLs than the beta isoform and is specifically expressed in OLs among the neural lineage [26], [38]. However, it is conceivable that PDGFR-β plays a role in OL differentiation, since PDGFR-β overexpression has been demonstrated in oligodendroglioma proliferation [50], [51]. Because of its low, but nonzero expression in OLs, the role of PDGFR-β and its interaction with the alpha subunit has not been studied in the OL differentiation process so far, however, potential links between SNX6 and PDGFR-β can be inferred from literature: (*i*) SNX6 is highly expressed in OLs and its expression increases with OPC differentiation [38] (*ii*) SNX6 has been shown to interact with several receptor tyrosine kinases, including PDGFR-β [52] (*iii*) SNX6 has been demonstrated to be part of the retromer complex implicated in endocytosis [53], [54], [55] along with other sorting nexin like Snx25 and Snx27, which are involved in endocytic trafficking of signalling receptors [56], [57] and to activate enzymes, like GIT1 (GrK2 interacting protein-1), implicated in G protein-coupled receptor desensitization, endocytosis and degradation [58]. (*iv*) GrK2 has been shown to be implicated in a retrofeedback loop leading to PDGFR-β desensitization through inter-phosphorylation events [59], [60], [61]. A controlling mechanism involving PDGFR-β, SNX6 and Dusp15/VHY during OL differentiation can thus be hypothesized. In addition, Dusp15 is a myristoylated protein, resulting in subcellular localization close to the plasmic membrane [41]. Myristoylation has also been described for Jsp1 (dusp22), a VH-related DUSP related to to dusp15/VHY, as being required for its activity [62]. By analogy this subcellular localization may be critical for Dsup15 in the context of modulating RTKs such as PDGFR-β. Finally, the PDGFR ligand PDGFBB is overexpressed in Schwann cells following peripheral nerve injury [63], which also results in enhanced PDGFR-β phosphorylation [64]. Given that PDGFBB is a trophic factor for neurons [65], [66], this system was suggested to be strongly associated with axonal regeneration. Our data suggest that this regulation could occur in the CNS during remyelination by OLs.

### Dusp15/VHY is Regulated in Demyelinating Diseases

Most of PTPs that are highly modulated in secondary MS patients or in MOG-induced EAE mice have been linked with inflammatory processes [27] (e.g. Ptpn22, CD45, PAC-1) but none of these proteins has been associated with OL signaling except Ptprr/PTP-SL, which has recently been shown to be a potential negative regulator of OL differentiation [26], and Ptpn6/SHP-1 a positive regulator [67]. Dusp15/VHY was among the ten most highly modulated PTPs in both MS patients and EAE samples. As Dusp15/VHY is to our knowledge not expressed in immune cells (BioGPS database [68]), we can deduce that the modulation occurs in other cell types, probably OLs. The context in which Dusp15/VHY is expressed in MS lesions together with the finding that Dusp15/VHY silencing results in OL differentiation *in vitro* suggests that Dusp15/VHY is a pharmacological target for promoting remyelination in multiple sclerosis.

## Materials and Methods

### Ethics Statement

All the experiments involving animals were performed in accordance with the Swiss regulations for animal welfare. All experimental protocols were submitted and approved by local veterinarian authorities (Geneva veterinarian office), the Geneva ethical commission and the local MerckSerono ethical committee (Licence number: 31.1.1040/3020-1/3). Experiments are subject to regular audit for animal welfare and material procedure compliance.

All research involving human tissues in the current study was approved by the Cleveland Clinic’s Institutional Review Board (IRB). Written consent was obtained from all the MS patients pre-mortem or from the next-of-kin post-mortem. Control tissues were procured from patients for whom there was consent by the next-of-kin for complete autopsy. The IRB determined that additional consent for use of redundant tissues from these cases for research could be waived.

### Oli-neu Cells Maintenance and Differentiation

Oli-neu cells were developed in the laboratory of Pr. Jacqueline Trotter from the Gutenberg university in Mainz [32]. and were kindly provided by her. Oli-neu cells were maintained in 75 cm^2^ or 150 cm^2^ flasks at 37°C in a humidified atmosphere containing 10% CO_2_ in Dulbecco Modified Eagle Medium (DMEM)–F-12 (Invitrogen #21331) supplemented with 50 µg/mL of apo-transferrin (Sigma T-1147), 5 ng/mL of sodium selenite (Sigma S-9133), 5 µg/mL of insulin (Sigma I-0516), 100 µM putrescine (Sigma P-7505), 22 nM progesterone (Sigma P-7556), 0.5 nM Tri-iodotyronine (Sigma T-6397), 1% horse serum (Invitrogen #26050), 100 U/mL penicillin-streptomycin (Invitrogen #15070) and 4 mM glutamine (Invitrogen #25030). Oli-neu were allowed to differentiate after plating in polypropylene multiwell plates coated or not with poly-D-lysine in a modified medium containing DMEM–F-12 supplemented with 50 µg/mL of apo-transferrin, 5 ng/mL of sodium selenite, 100 µM putrescine, 130 nM progesterone, 200 nM Tri-iodotyronine, 1% horse serum, 1% penicillin-streptomycin, and 4 mM glutamine. When chemically induced, Oli-neu differentiation was obtained by using the ErbB2 inhibitor PD174265 at 1 uM (Alexis ALX-270-323).

### RNA Extraction and Reverse Transcription

RNAs were extracted using the Rneasy minikit Plus (Qiagen) following the supplier instructions. Briefly, a lysis buffer (350 µL/well of 6-well plate) allowed cell components extraction. Samples were clarified using the Qiashredder (Qiagen) filtration system. At the end of the extraction procedure, RNAs were eluted by addition of 30 µL RNAse-free water. Concentration of RNAs samples were measured using a nanodrop bioanalyzer and overall quality was assessed by measurement of 260/280 nm *ratio*. A total amount of 1 µg of RNA was used as template for reverse transcription using the quantascript kit (#95048-100, Quanta Biosciences) containing a pre-mixture of oligo(dT)20 (Invitrogen #18418), reverse transcriptase Superscript III (Invitrogen #18080), and random primers (Invitrogen #48190) or the iscript kit (Biorad). Mixture was cycled following the procedure: i) 5 min at 25°C, ii) 30 min at 42°C, iii) 5 min at 85°C, iv) 10°C for ever.

### Real Time Quantitative PCR Analysis

cDNAs were synthesized from the previously extracted RNA library and diluted 50 times. Real-time PCR was performed in 384-well plate format, each well containing a mixture of 2.5 µL of the pre-mixed primer pair at 1.2 µM, 2.5 µL of 50 times diluted cDNA and 5 µL of SYBR Green PCR master mix (Quantifast, Qiagen #204054). Each reaction was run in triplicates in a 7900HT Fast real-Time PCR system (Applied Biosystems) following the same cycling protocols described above (i to iv). Primers specificity and primers efficacy were previously verified by performing dissociation curves and a range of cDNA dilutions respectively (slope analysis). The primers selected are listed below. The primers corresponding to the mouse and human PTP set are listed in Table S1). Sequences corresponding to non PTP oligonucleotides are listed in Table 6. Analysis was performed using the classical delta Ct method. Statistical analyses were performed using paired *t*-test, given the low number of individuals/animals within groups.

**Table 6:** Oligonucleotides sequences.

Primer	Supplier	Reference, sequence or region
MBP	Eurogentech	Exon6; Fwd: GCAGAAGCCAGGATTTGGC; Rev: CCCCTTGAATCCCTTGTGAG
CNP	Eurogentech	Fwd: TAACCCTCCCTTAGCCCCTG; Rev: TCCCTAGCATGTGGCAGCT
PLP	Eurogentech	Fwd: GGCTAGGACATCCCGACAAG; Rev: GCAAACACCAGGAGCCAT
Cspg4	Qiagen	QT00120407
ErBb2	Qiagen	QT01074402
VHY/Dusp15	Qiagen	QT00171605
VHY/Dusp15	Thermoscientific	Fwd: GTGATCGCCTATGTGATG; Rev: AAAGCCCGGGTTTGGGTTGG
Glepp1/Ptpro	Qiagen	QT00134540
STEP/Ptpn5	Qiagen	QT01063580
PTP-SL/Ptprr	Qiagen	QT00173530
PTPδ/Ptprd	Qiagen	QT01167180
MKP-2/Dusp4	Qiagen	QT00140357
MKP-3/Dusp6	Qiagen	QT00101997
PTPBAS/Ptpn13	Qiagen	QT00097244
TENSIN-3/TNS3	Qiagen	QT01557437
TC-PTP/Ptpn2	Qiagen	QT01063573
Actin	Thermoscientific	Fwd: AACCCTAAGGCCAACCGTGARev: GCCTGGATGGCTACGTACATG
Ubc	Qiagen	QT00245189
Psmb3	Qiagen	QT00103649
Hbms	Proligo	3R/3F; 294S433-F/2946440-F

### Immunocytochemistry

Oli-neu ells were cultured, treated and/or transfected as described and fixed with a 1% glutaraldehyde (Fluka #49631) solution in 1xPBS for 15 min. Cells were then washed twice with 1xPBS and permeabilized/blocked for at least 1hour with a PBS^+^ solution containing 10% calf serum and 0.1% TritonX-100 in 1xPBS. 100 µL of the primary antibody (see references below) solution diluted in PBS^+^ at the desired concentration were added per well of 96-well plate and cells were incubated overnight at 4°C under slow rotation. Cells were then washed four times with 1xPBS and 100 µL of a secondary antibody (see references below) solution diluted at 1∶500 in PBS^+^ were added per well. Plates were incubated for 2 hours at room temperature under slow rotation. Each well was then washed four times with 1xPBS prior to further analyses, ie morphological assessment or MBP *in-cell* ELISA.

### MBP *in-cell* ELISA

Cells were cultured in poly-*D*-lysine-coated 96-well plates (BD Biocoat, #356461**)**, fixed and immunostained as described using a goat anti-MBP primary antibody (Santa Cruz Biotechnologies, sc-13914, 1∶500) and a secondary anti-goat HRP-coupled antibody (Santa Cruz Biotechnology, sc-2020, 1∶500). Then, were added 100 µL of a solution containing the HRP chromogenic substrate ABTS (2,2′-azino-bis-(3-ethylbenzothiazolin-6-sulfonic acid) diammonium salt, Sigma, #A1888) at 0.2 mg/mL and H_2_O_2_ at 0.02% in a 7 mM citrate buffer at pH 4.5. After 2 hours incubation at room temperature under rotation, optical density was measured with an absorbance spectrophotometer at 405 nm. Cells were then washed twice with PBS and 200 µL/well of a protein titration solution containing bicinchroninic acid (Pierce BCA Protein Assay Kit, #23225, Thermoscientific) were added. Plates were incubated for 2 hours at room temperature and optical density was red at 620 nm allowing total protein content measurement. MBP levels were given by the *ratio* 405 nm/620 nm and relatively expressed in percentage of undifferentiated controls as the Mean ± SEM of at least 3 experiments in technical quadruplicates.

### Morphological Measurements

Oli-neu were cultured at a density of 3000 cells per well in poly-*D*-lysine-coated 96-well plates (BD Biocoat, #356461), fixed and immunostained as described above using a monoclonal mouse anti-A2B5 primary antibody (Sigma, A8229-100UG, 1∶500) and a secondary goat anti-mouse IgG Cyanin3-coupled antibody (Jackson Immunoresearch, 115-165-166, 1∶500). A staining of nuclei was performed by addition of 100 µL of a solution of HOECHST 33342 (#H3570, Molecular probes, Invitrogen) at 1∶1000 for 2 minutes at room temperature followed by a washing step with PBS. Morphological analysis was assessed using an automated platform Cellomics ArrayScan HCS reader developed by Thermoscientific, performing image acquisition (magnification x10; ≥200 cells per well; ≥20 fields) and fluorescent pixels monitoring to allow for subsequent parameters analyses including total process area per cell (area represented by all processes surrounding cell bodies excluding cell bodies areas and normalized by cell number). Results are expressed in % of undifferentiated controls as the Mean ± SEM of at least 3 experiments in technical quadruplicates.

### Western Blot Analysis

Cells were cultured at a minimal density of 5×10^4^ cells/cm^2^, washed once with PBS then lysed with a RIPA lysis buffer (10–20 µL/cm^2^) containing 50 mM Tris-HCl. 1% TX-100, 0.2% SDS, 0.5% NP-40, 150 mM NaCl supplemented with protease inhibitors (Roche, #11697498001). Protein contents were then measured using a protein titration solution containing bicinchroninic acid (Pierce BCA Protein Assay Kit, #23225, Thermoscientific). 10–20 µg of protein samples were mixed with LDS loading buffer (NuPAGE, NP0007, Invitrogen) containing 0.5 mM Dithiothreitol (P/N y00147, Invitrogen) and heated at 100°C for 2 minutes. Samples were then loaded in Sodium dodecyl sulphate (SDS) polyacrylamide gels (Invitrogen NuPAGE Novex Bis-Tris gels,4–12%, NP0336BOX) and run in MES SDS buffer (NuPAGE, NP0002, Invitrogen) according to the manufacturer’s instructions. Proteins were then transferred to nitrocellulose membrane (Invitrogen LC2001) in transfer buffer (NP0006-1, Invitrogen) containing 20% of methanol using a semidry transfer apparatus (Hoefer SemiPhor; Amersham Pharmacia Biotech). The membrane was blocked using a 10% solution of non fat milk in a PBS-Tween20 0.2% washing buffer. Membranes were incubated overnight at 4°C in a solution of anti-CNP ((M-300) Santa Cruz sc-30158) rabbit polyclonal antibody or anti-MBP ((C-16) Santa Cruz sc-13914) goat polyclonal antibody or anti-tubulin-beta ((H-235) Santa Cruz sc-9104) rabbit polyclonal antibody, all at a 1∶1,000 dilution. Membranes were then washed six times with PBS-Tween20 0.2% and incubated for 1 h at room temperature with a solution of horseradish peroxidase (HRP)-conjugated antiserum (goat anti-rabbit Ig-HRP [Bio-Rad 170-6515] or donkey antigoat Ig-HRP [Santa Cruz sc-2020], all at 1∶2,000 dilution). Four additional washes were performed before exposure to the chemoluminescent substrate (ECL kit; Amersham Pharmacia Biotech RPN 2106).

### Mixed Cortical Cultures

All the experiments were performed in accordance with the Swiss regulations for animal welfare. Mouse mixed cortical cultures were performed as previously described [69], [70]. E16 mouse embryos were obtained from pregnant NMRI female euthanized under CO_2_ and placed immediately in cold dissection medium (HBSS supplemented with 100 U/mL penicillin/streptomycin (Invitrogen 15070-063), 0.075% of sodium bicarbonate (Gibco, 25080-06) and 10 mM HEPES (Gibco, 15630)). Cortices were dissected after meninges removal and transferred into a 50 mL Falcon™ tube containing 10 mL of dissection medium. 100 µL of trypsin were added and the mixture was incubated for 20 min at 37°C. Trypsin was then inactivated by addition of 200 µL Fetal Calf Serum (FCS, Invitrogen, 10108-165) and tissues were mechanically dissociated by consecutive pipetting. The suspension was then centrifuged at 1300 rpm at 4°C for 10 min. The supernatant was removed and the pellet was resuspended in culture medium prewarmed at 37°C (DMEM (Gibco, 31966), Transferrin (Sigma, P-2252) at 100 µg/mL, putrescine (Sigma, P-7505) at 16.1 µg/mL, insulin (Sigma, I-882) at 9.3 µg/mL, BSA (Gibco, 15260-037) at 10.7 µg/mL, progesterone (Sigma, P-7556) at 62 ng/mL, triiodothyronin (Sigma, T-5516) at 301 ng/mL, tetraiodothyronin (Sigma, T-0397) at 400 ng/mL, sodium selenite (Sigma, S-9133) at 39 ng/mL, glutamine (Invitrogen, 25030-024) at 2 mM, penicillin/streptomycin (Invitrogen, 15070-063) at 100 U/mL and FCS (Invitrogen, 10108-165) at 0.5%). The suspension was centrifuged at 1300 rpm for 10 min. The supernatant was discarded and the pellet was resuspended at 10x6 cells/mL, and dispensed in poly-D-lysine coated plates (100 µL/well) and maintained for 2 weeks at 37°C in a humidified atmosphere enriched with 10% CO_2_. 150 µL of 200 µL were refreshed at DIV2, 5, 7, 9, 12 and 15.

### Experimental Autoimmune Encephalomyelitis Inoculation

All the experiments were performed in accordance with the Swiss regulations for animal welfare. All animals were supplied with food and water ad libitum and maintained on a 12 h light/dark schedule in a temperature and humidity-controlled environment. 9 to 10 weeks old female mice (C57/BL6, Elevage Janvier) were immunized by subcutaneous injection of 200 µg of MOG peptide (MOG 35-55 MEVGWYRSPFSRVVHLYRNGK, NeoMPS) in complete Freund’s adjuvant (CFA) containing 250 µg per mouse of Mycobacterium tuberculosis (H37A; DIFCO). A total of 300 ng/mouse of Pertussis toxin (Alexis) was administrated by intraperitoneal (ip) route the day of the immunization as well as two days later.

Mice were observed daily, weighted and neurological signs were quantified using an arbitrary 10-points clinical scale to assess paralysis (tail, hind limbs and fore limbs), incontinency and death (Table 6).

For RNA extraction, animals were sacrified with CO_2_, blood was collected by intracardiac ponction and cerebellum and spinal cord were removed, frozen in liquid nitrogen and kept at −80°C until RNA preparation.

### Silencing RNA Transfection

Oli-neu cells were transfected with annealed small interfering RNAs (si-RNAs) duplexes at 10 nM using the HiPerfect transfection reagent (Qiagen). Transfection complexes were prepared in OptiMEM medium (Invitrogen catalog no.51985) for 10 min at room temperature according to the manufacturer’s instructions. Percentages of knock-down (KD%) were measured by q-PCR analysis as described earlier. For each experiment, negative controls used as reference were (i) a si-RNA targeting luciferase and (ii) a MOCK control with transfection reagent only. An ErbB2 si-RNA mixture was used as previously described ss positive control for differentiation [26].

### *In vitro* Dephosphorylation Assay

Recombinant full length Nterm GST-Dusp15/VHY was purchased from AbNova (H00128853-P01) with specifications regarding the use of phosphate-free elution buffers. A dilution range of the recombinant enzyme diluted in buffer containing 20 mM Tris-HCl, 0.06% NP-40 and 1 mM dithiothreitol at pH6, 7, 8 and 9 was performed in 96-well plates. To 45 µL of enzyme were added 5 µL of a solution of DiFMUP at 50 µM in water (6,8-difluoro-4-methyumbelliferyl phosphate, #D6567, Molecular probes). Plates were incubated in darkness and fluorescence intensity was monitored at 355/460 nm at different time points and overnight on a spectrofluorimeter. Absence of enzyme served as negative control. For phospho-peptide substrates determination, 384-well plates containing targeted phospho-peptides P-Tyr and P-Ser/P-Thr (JPT, Phosphatase substrates sets) were used. To 5 µL of phospho-peptide at 500 nM were added 15 µL of a solution of recombinant Dusp15/VHY at 2.5 ng/µL diluted in assay buffer at pH6. Plates were incubated overnight and 20 µL/well of malachite green (Nalgene, AK-111) were added. Optical density was monitored using a UV spectrophotometer at 620 nm. Wells containing only the enzyme dilution were used as negative control. This value was used to normalize the dataset. As a non specific phosphatase substrate, DiFMUP was used as positive control of dephosphorylation. In these conditions, malachite green is able to trap the free phosphate as for any phospho-peptide leading to a green coloration.

## Supporting Information

Table S1 Validated human and mouse oligonucleotides set for q-PCR quantification. Set of primers selected for the q-PCR based PTP expression profile.
(PDF)

Table S2 Differential gene expression of PTP family members in MS white matter lesions.
(PDF)

Table S3 Differential gene expression of PTP family members in MS grey matter lesions.
(PDF)

Table S4 PTP gene expression in spinal cord and cerebellum from MOG-induced EAE mice. Differential gene expression of PTP family members in EAE spinal cord and cerebellum lesions.
(PDF)

Table S5 PTP gene expression during OL differentiation in mouse mixed cortical cultures. Differential gene expression of PTP family members in mixed cortical culture undergoing OL differentiation.
(PDF)
