# Supplementary material for: Identification of VHY/Dusp15 as a Regulator of Oligodendrocyte Differentiation through a Systematic Genomics Approach
Source: PLoS One. 2012 Jul 11;7(7):e40457. doi: 10.1371/journal.pone.0040457 (PMC3394735; doi:10.1371/journal.pone.0040457)
Supplement: Table S1 — Validated human and mouse oligonucleotides set for q-PCR quantification. Set of primers selected for the q-PCR based PTP expression profile. (PDF) [file pone.0040457.s001.pdf]

Table S1. *Validated human and mouse oligonucleotides set for q-PCR quantification*

Set of primers selected for the q-PCR based PTP expression profile

**Mouse phosphatases primer set**				**Human phosphatases primer set**			
**Synonym**	**Gene**	**Reference**	**Supplier**	**Name**	**Gene**	**Reference**	**Supplier**
MKP1	*Dusp1*	QT00288638	Qiagen	MKP1	*Dusp1*	QT00036638	Qiagen
MKP2	*Dusp4*	QT00140357	Qiagen	MKP2	*Dusp4*	PPH00058A-200	SABiosciences
hVH3	*Dusp5*	QT01065617	Qiagen	hVH3	*Dusp5*	QT00001337	Qiagen
PAC1	*Dusp2*	QT00248283	Qiagen	PAC1	*Dusp2*	PPH00049A-200	SABiosciences
MKP3	*Dusp6*	QT00101997	Qiagen	MKP3	*Dusp6*	QT00209986	Qiagen
PYST2	*Dusp7*	QT00155337	Qiagen	PYST2	*Dusp7*	QT00001988	Qiagen
MKP5	*Dusp10*	QT00152257	Qiagen	MKP4	*Dusp9*	QT00207326	Qiagen
hVH5	*Dusp8*	QT00118650	Qiagen	MKP5	*Dusp10*	QT00082117	Qiagen
MKP7	*Dusp16*	QT00097272	Qiagen	hVH5	*Dusp8*	QT00009212	Qiagen
MK-Styx	*Styxl1*	PPM34884A-200	SABiosciences	MKP7	*Dusp16*	QT00094178	Qiagen
VHR	*Dusp3*	QT00138467	Qiagen	MK-Styx	*Styxl1*	QT00004032	Qiagen
MKP8	*Dusp26*	QT00112630	Qiagen	VHR	*Dusp3*	QT00030107	Qiagen
MKP6	*Dusp14*	QT00284424	Qiagen	MKP6	*Dusp14*	QT00203735	Qiagen
LMW DSP20	*Dusp18*	QT00119322	Qiagen	LMW DSP20	*Dusp18*	QT00090202	Qiagen
SKRP1	*Dusp19*	QT00169624	Qiagen	SKRP1	*Dusp19*	QT00023275	Qiagen
Styx	*Styx*	QT01531992	Qiagen	Styx	*Styx*	QT00057330	Qiagen
JSP1	*Dusp22*	QT00158431	Qiagen	JSP1	*Dusp22*	QT01023582	Qiagen
VHY	*Dusp15*	QT00171605	Qiagen	VHY	*Dusp15*	PPH18348A-200	SABiosciences
SSH1	*Ssh1*	QT00165011	Qiagen	SSH1	*Ssh1*	QT00011417	Qiagen
SSH2	*Ssh2*	QT00101199	Qiagen	SSH2	*Ssh2*	QT00054852	Qiagen
SSH3	*Ssh3*	QT00150227	Qiagen	SSH3	*Ssh3*	QT00007644	Qiagen
HYVH1	*Dusp12*	QT00124600	Qiagen	HYVH1	*Dusp12*	QT00060319	Qiagen
PTEN	*Pten*	QT00141568	Qiagen	PTEN	*Pten*	QT01676969	Qiagen
PRL1	*Ptp4a1*	QT00156464	Qiagen	PRL1	*Ptp4A1*	QT00028203	Qiagen
PRL2	*Ptp4a2*	QT00143332	Qiagen	PRL2	*Ptp4A2*	QT00080290	Qiagen
PRL3	*Ptp4a3*	QT00138243	Qiagen	PRL3	*Ptp4A3*	QT00009471	Qiagen
cdc14a	*Cdc14a*	QT01051764	Qiagen	cdc14a	*Cdc14A*	QT00094976	Qiagen
cdc14b	*Cdc14b*	QT01079148	Qiagen	cdc14b	*Cdc14B*	QT00009107	Qiagen
PIR1	*Dusp11*	QT00104867	Qiagen	PIR1	*Dusp11*	QT00004753	Qiagen
MCE	*Rngtt*	QT00151564	Qiagen	MCE	*Rngtt*	QT00021623	Qiagen
LAFORIN	*Epm2a*	QT00261842	Qiagen	LAFORIN	*Epm2A*	PPH22251A-200	SABiosciences
PTP 9q22	*Ptpdc1*	QT00161973	Qiagen	PTP 9q22	*Ptpdc1*	QT00061663	Qiagen
KAP1	*Cdkn3*	QT01561763	Qiagen	KAP1	*Cdkn3*	QT00014728	Qiagen
PTP MT1	*Ptpmt1*	QT00114772	Qiagen	PTP MT1	*Ptpmt1*	QT01029448	Qiagen
VHZ	*Dusp23*	QT01075487	Qiagen	VHZ	*Dusp23*	QT00054313	Qiagen
MTM1	*Mtm1*	QT01066079	Qiagen	MTM1	*Mtm1*	QT00061565	Qiagen
MTMR1	*Mtmr1*	QT00147070	Qiagen	MTMR1	*Mtmr1*	QT00041545	Qiagen
MTMR2	*Mtmr2*	QT00144795	Qiagen	MTMR2	*Mtmr2*	QT00034111	Qiagen
MTMR3	*Mtmr3*	QT01068655	Qiagen	MTMR3	*Mtmr3*	QT00075488	Qiagen
MTMR4	*Mtmr4*	QT00148757	Qiagen	MTMR4	*Mtmr4*	QT00082523	Qiagen
MTMR6	*Mtmr6*	QT00147994	Qiagen	MTMR6	*Mtmr6*	QT00076125	Qiagen
MTMR7	*Mtmr7*	QT00175868	Qiagen	MTMR7	*Mtmr7*	QT00197519	Qiagen
MTMR9	*Mtmr9*	QT00102305	Qiagen	MTMR8	*Mtmr8*	QT00059535	Qiagen
MTMR11	*Mtmr11*	QT00157318	Qiagen	MTMR9	*Mtmr9*	QT00085421	Qiagen
MTMR12	*Mtmr12*	QT00132384	Qiagen	MTMR11	*Mtmr11*	QT00063742	Qiagen
MTMR10	*Mtmr10*	QT01067675	Qiagen	MTMR12	*Mtmr12*	QT00081830	Qiagen
MTMR5	*Sbf1*	QT01549450	Qiagen	MTMR10	*Mtmr10*	QT00007833	Qiagen
MTMR13	*Sbf2*	QT00125951	Qiagen	MTMR5	*Sbf1*	PPH11198E-200	SABiosciences
TENSIN-1	*Tns1*	PPM33341A-200	SABiosciences	MTMR13	*Sbf2*	QT00056035	Qiagen

TENSIN-3	*Tns3*	QT01557437	Qiagen	TENSIN-1	*Tns1*	QT00081627	Qiagen
TENSIN-2	*Tenc1*	QT01066800	Qiagen	TENSIN-3	*Tns3*	QT00081396	Qiagen
cdc25a	*Cdc25a*	QT01058778	Qiagen	TENSIN-2	*Tenc1*	QT00002548	Qiagen
cdc25b	*Cdc25b*	QT00127806	Qiagen	cdc25a	*Cdc25A*	QT00001078	Qiagen
cdc25c	*Cdc25c*	QT01055222	Qiagen	cdc25b	*Cdc25B*	QT00028350	Qiagen
PTP1B	*Ptpn1*	QT00166418	Qiagen	cdc25c	*Cdc25C*	QT00000350	Qiagen
TCPTP	*Ptpn2*	QT01063573	Qiagen	PTP1B	*Ptpn1*	QT01006978	Qiagen
SHP1	*Ptpn6*	QT00155967	Qiagen	TCPTP	*Ptpn2*	PPH21327A-200	SABiosciences
SHP2	*Ptpn11*	QT00103362	Qiagen	SHP1	*Ptpn6*	QT00011725	Qiagen
MEG2	*Ptpn9*	QT00170520	Qiagen	SHP2	*Ptpn11*	QT00070336	Qiagen
PEST	*Ptpn12*	QT00168126	Qiagen	MEG2	*Ptpn9*	QT00068579	Qiagen
LyPTP	*Ptpn22*	QT00103943	Qiagen	PEST	*Ptpn12*	QT00011557	Qiagen
BDP1	*Ptpn18*	QT00097027	Qiagen	LyPTP	*Ptpn22*	PPH10382A-200	SABiosciences
MEG1	*Ptpn4*	PPM35219A-200	SABiosciences	BDP1	*Ptpn18*	QT00089257	Qiagen
PTPH1	*Ptpn3*	QT00264649	Qiagen	MEG1	*Ptpn4*	QT00076503	Qiagen
PTPD1	*Ptpn21*	QT00197862	Qiagen	PTPH1	*Ptpn3*	QT00067249	Qiagen
PEZ	*Ptpn14*	QT00107674	Qiagen	PTPD1	*Ptpn21*	QT00065100	Qiagen
PTPBAS	*Ptpn13*	QT00097244	Qiagen	PEZ	*Ptpn14*	QT00005600	Qiagen
PTPTyp	*Ptpn20*	PPM25464A-200	SABiosciences	PTPBAS	*Ptpn13*	QT00054446	Qiagen
HDPTP	*Ptpn23*	QT01560692	Qiagen	HDPTP	*Ptpn23*	PPH08613B-200	SABiosciences
HePTP	*Ptpn7*	QT01073611	Qiagen	HePTP	*Ptpn7*	QT00063413	Qiagen
CD45	*Ptprc*	QT00139405	Qiagen	CD45	*Ptprc*	QT00028791	Qiagen
LAR	*Ptprf*	PPM05105E-200	SABiosciences	LAR	*Ptprf*	QT00041062	Qiagen
PTPdelta	*Ptprd*	QT01167180	Qiagen	PTPdelta	*Ptprd*	QT00059640	Qiagen
PTPsigma	*Ptprs*	QT00150416	Qiagen	PTPsigma	*Ptprs*	QT00063504	Qiagen
PTPkappa	*Ptprk*	QT01063615	Qiagen	PTPkappa	*Ptprk*	QT00031080	Qiagen
PTPmu	*Ptprm*	QT00167545	Qiagen	PTPmu	*Ptprm*	QT00010199	Qiagen
PTPrho	*Ptprt*	QT01063594	Qiagen	PTPrho	*Ptprt*	QT00097923	Qiagen
PTPS31	*Ptprq*	QT01077237	Qiagen	PTPlambda	*Ptpru*	QT00005677	Qiagen
GLEPP1	*Ptpro*	QT00134540	Qiagen	PTPS31	*Ptprq*	QT01150597	Qiagen
PTPbeta	*Ptprb*	QT00197981	Qiagen	GLEPP1	*Ptpro*	QT00037695	Qiagen
DEP1	*Ptprj*	QT00169785	Qiagen	PTPbeta	*Ptprb*	QT00054411	Qiagen
SAP1	*Ptprh*	QT01059415	Qiagen	DEP1	*Ptprj*	QT00043876	Qiagen
PTPalpha	*Ptpra*	QT00141610	Qiagen	SAP1	*Ptprh*	PPH13725A-200	SABiosciences
PTPepsilon	*Ptpre*	QT00101941	Qiagen	PTPalpha	*Ptpra*	QT00494480	Qiagen
PTPgamma	*Ptprg*	QT00160832	Qiagen	PTPepsilon	*Ptpre*	QT00021567	Qiagen
PTPzeta	*Ptprz1*	QT01057854	Qiagen	PTPgamma	*Ptprg*	QT00060116	Qiagen
PCPTP1	*Ptprr*	QT00173530	Qiagen	PTPzeta	*Ptprz1*	QT00083300	Qiagen
STEP	*Ptpn5*	QT01063580	Qiagen	PCPTP1	*Ptprr*	PPH14225A-200	SABiosciences
IA2	*Ptprn*	QT01063608	Qiagen	STEP	*Ptpn5*	QT00013909	Qiagen
IA2beta	*Ptprn2*	QT01057868	Qiagen	IA2	*Ptprn*	QT00015421	Qiagen
PTPESP	*Ptprv*	QT01070930	Qiagen	IA2beta	*Ptprn2*	QT00074375	Qiagen
LMW PTP	*Acp1*	QT00135135	Qiagen	LMW PTP	*Acp1*	QT00022820	Qiagen
