# Supplementary material for: Identification of VHY/Dusp15 as a Regulator of Oligodendrocyte Differentiation through a Systematic Genomics Approach
Source: PLoS One. 2012 Jul 11;7(7):e40457. doi: 10.1371/journal.pone.0040457 (PMC3394735; doi:10.1371/journal.pone.0040457)
Supplement: Table S2 — Differential gene expression of PTP family members in MS white matter lesions. (PDF) [file pone.0040457.s002.pdf]

Table S2. Differential gene expression of PTP family members in MS white matter lesions

	WML vs CWM			WML vs NAWM			NAWM vs CWM	
	Fold	Pvalue		Fold	Pvalue		Fold	Pvalue
**PTPN18**	-15.47	0.004	**PTPN18**	-14.15	0.019	**DUSP2**	-3.30	0.009
**DUSP1**	-6.97	0.001	**DUSP1**	-5.82	0.000	**DUSP6**	-2.67	0.003
**PTP4A2**	-5.82	0.001	**MTMR10**	-5.30	0.002	**PTPN7**	-2.18	0.115
**SSH3**	-5.46	0.007	**SSH3**	-4.87	0.001	**PTPN22**	-2.11	0.032
**DUSP22**	-4.77	0.002	**DUSP22**	-3.93	0.002	**PTPN1**	-1.97	0.141
**MTMR7**	-4.51	0.002	**DUSP11**	-3.70	0.013	**PTP4A2**	-1.79	0.002
**PTPRD**	-4.12	0.001	**PTP4A2**	-3.25	0.001	**PTPRE**	-1.68	0.022
**MTMR10**	-4.08	0.000	**PTPN14**	-3.03	0.042	**PTPN6**	-1.67	0.044
**DUSP11**	-3.71	0.006	**PTPRD**	-3.02	0.003	**DUSP5**	-1.65	0.008
**DUSP5**	-3.69	0.016	**MTMR2**	-2.86	0.016	**MTMR7**	-1.61	0.010
**PTPN13**	-3.55	0.011	**MTMR7**	-2.80	0.002	**PTPRC**	-1.60	0.090
**PTPN14**	-3.31	0.008	**PTPRZ1**	-2.63	0.008	**MTMR8**	-1.57	0.089
**PTPN1**	-3.18	0.133	**DUSP3**	-2.62	0.004	**PTPRH**	-1.56	0.097
**PTPN11**	-3.16	0.001	**MTMR12**	-2.60	0.003	**DUSP15**	-1.54	0.048
**MTM1**	-2.69	0.000	**MTM1**	-2.42	0.037	**PTP4A3**	-1.53	0.006
**PTPRC**	-2.67	0.096	**PTPN11**	-2.38	0.002	**PTPN13**	-1.50	0.071
**PTPRZ1**	-2.59	0.001	**PTPN13**	-2.37	0.005	**PTPRQ2**	-1.48	0.128
**SSH2**	-2.47	0.014	**PTPRU**	-2.25	0.046	**MTMR11**	-1.45	0.075
**MTMR12**	-2.43	0.004	**DUSP5**	-2.24	0.032	**hEPM2A**	-1.38	0.051
**MTMR2**	-2.39	0.007	**PTPRJ**	-2.23	0.053	**PTPRD**	-1.36	0.019
**DUSP16**	-2.34	0.012	**PTPRF**	-2.20	0.007	**PTPN11**	-1.33	0.012
**PTP4A1**	-2.27	0.013	**DUSP16**	-2.03	0.025	**PTPDC1**	-1.30	0.026
**PTPRF**	-2.08	0.012	**SSH2**	-2.00	0.018	**hRNGTT**	-1.27	0.006
**PTPRU**	-1.92	0.072	**DUSP10**	-1.93	0.031	**SSH2**	-1.23	0.009
**PTPRJ**	-1.92	0.037	**PTP4A1**	-1.90	0.032	**CDC14b**	-1.23	0.186
**TNS1**	-1.88	0.010	**TNS1**	-1.79	0.003	**CDKN3**	-1.23	0.328
**DUSP3**	-1.82	0.005	**PTPRC**	-1.67	0.105	**PTPMT1**	-1.22	0.020
**DUSP10**	-1.76	0.060	**MTMR4**	-1.67	0.017	**DUSP22**	-1.21	0.052
**SBF2**	-1.71	0.003	**PTPRK**	-1.65	0.024	**DUSP1**	-1.20	0.016
**MTMR4**	-1.69	0.004	**PTPN1**	-1.61	0.112	**PTP4A1**	-1.20	0.074
**PTPMT1**	-1.54	0.009	**SBF2**	-1.60	0.021	**PTPN12**	-1.19	0.165
**PTPRA**	-1.54	0.000	**DUSP7**	-1.57	0.017	**DUSP9**	-1.17	0.271
**CDC14A**	-1.39	0.215	**PTPRA**	-1.49	0.007	**STYXL1**	-1.17	0.137
**PTPN4**	-1.35	0.119	**PTPN23**	-1.47	0.108	**CDC14A**	-1.16	0.154
**STYXL1**	-1.27	0.119	**PTPRB**	-1.43	0.121	**DUSP4**	-1.15	0.210
**PTPRK**	-1.24	0.052	**PTPN4**	-1.36	0.051	**DUSP16**	-1.15	0.231
**MTMR6**	-1.22	0.282	**STYX**	-1.29	0.018	**TENC1**	-1.15	0.079
**DUSP7**	-1.19	0.164	**MTMR6**	-1.29	0.146	**SSH3**	-1.12	0.190
**PTPRE**	-1.17	0.123	**PTPMT1**	-1.27	0.036	**MTM1**	-1.11	0.287
**PTPDC1**	-1.15	0.047	**PTPRO**	-1.23	0.122	**PTPN14**	-1.09	0.246
**STYX**	-1.11	0.008	**MTMR1**	-1.22	0.052	**PTPN18**	-1.09	0.371
**MTMR1**	-1.08	0.362	**CDC14A**	-1.20	0.272	**DUSP12**	-1.07	0.263
**PTPRB**	-1.07	0.408	**SSH1**	-1.13	0.181	**ACP1**	-1.07	0.315
**MTMR9**	-1.06	0.406	**STYXL1**	-1.09	0.301	**SBF2**	-1.07	0.192
**PTEN**	-1.02	0.467	**PTEN**	-1.08	0.117	**TNS1**	-1.05	0.206
**PTPN12**	1.04	0.411	**PTPN21**	-1.06	0.272	**CDC25A**	-1.05	0.471
**PTPRO**	1.10	0.280	**MTMR9**	-1.05	0.367	**PTPRA**	-1.04	0.203
**PTPRS**	1.17	0.126	**PTPRS**	1.00	0.481	**MTMR4**	-1.01	0.402
**SBF1**	1.19	0.144	**SBF1**	1.10	0.173	**MTMR9**	-1.01	0.474

**PTPN23**	1.20	0.184	**TNS3**	1.12	0.249	**DUSP11**	-1.00	0.488
**CDKN3**	1.23	0.298	**PTPDC1**	1.13	0.161	**PTPRG**	1.00	0.481
**PTPRG**	1.26	0.012	**PTPRN**	1.19	0.279	**PTPN4**	1.01	0.475
**SSH1**	1.28	0.151	**PTPN12**	1.24	0.066	**PTPRZ1**	1.01	0.411
**hRNGTT**	1.34	0.098	**PTPRG**	1.25	0.017	**PTPRM**	1.02	0.433
**CDC14b**	1.54	0.049	**PTPRE**	1.43	0.001	**MTMR3**	1.03	0.291
**PTPN21**	1.55	0.108	**CDKN3**	1.51	0.183	**MTMR6**	1.06	0.332
**TNS3**	1.72	0.078	**hRNGTT**	1.71	0.049	**PTPRF**	1.06	0.175
**MTMR3**	1.83	0.069	**MTMR3**	1.78	0.061	**PTEN**	1.06	0.343
**PTPRN**	1.90	0.053	**CDC25B**	1.85	0.021	**MTMR12**	1.07	0.279
**DUSP12**	2.29	0.015	**CDC14b**	1.89	0.153	**SBF1**	1.08	0.106
**CDC25B**	2.31	0.028	**PTPN9**	2.35	0.026	**DUSP10**	1.09	0.038
**CDC25A**	2.35	0.317	**DUSP12**	2.46	0.021	**PTPN2**	1.10	0.277
**TENC1**	2.57	0.004	**CDC25A**	2.46	0.194	**PTPN3**	1.12	0.077
**PTPN9**	2.92	0.015	**DUSP23**	2.72	0.030	**MTMR1**	1.13	0.309
**DUSP2**	3.07	0.051	**PTPRT**	2.76	0.034	**PTPRN2**	1.14	0.055
**hEPM2A**	3.08	0.011	**DUSP14**	2.78	0.007	**PTPRJ**	1.16	0.251
**DUSP14**	3.48	0.004	**TENC1**	2.96	0.004	**STYX**	1.16	0.030
**PTPN7**	3.61	0.321	**DUSP8**	3.18	0.012	**PTPRS**	1.16	0.107
**DUSP23**	4.07	0.020	**CDC25C**	3.21	0.224	**PTPRU**	1.17	0.061
**CDC25C**	4.38	0.102	**PTPN2**	4.17	0.004	**MTMR2**	1.20	0.063
**PTP4A3**	4.41	0.001	**hEPM2A**	4.26	0.005	**PTPN9**	1.24	0.048
**DUSP8**	4.47	0.017	**PTPN3**	4.73	0.003	**DUSP14**	1.25	0.055
**PTPN2**	4.57	0.007	**ACP1**	4.91	0.029	**CDC25B**	1.25	0.065
**ACP1**	4.58	0.034	**PTPN5**	4.97	0.005	**MTMR10**	1.30	0.017
**PTPN3**	5.29	0.002	**DUSP18**	5.19	0.034	**DUSP7**	1.33	0.026
**PTPRT**	5.52	0.021	**PTPRN2**	6.20	0.001	**PTPRK**	1.33	0.031
**DUSP6**	5.88	0.014	**PTP4A3**	6.77	0.000	**PTPRB**	1.35	0.006
**DUSP4**	6.13	0.011	**DUSP4**	7.07	0.008	**PTPRO**	1.36	0.013
**PTPRH**	6.58	0.008	**PTPRM**	7.34	0.006	**CDC25C**	1.36	0.499
**PTPRN2**	7.09	0.001	**PTPN7**	7.85	0.265	**DUSP8**	1.41	0.066
**PTPRM**	7.45	0.007	**PTPRR**	9.55	0.008	**DUSP3**	1.44	0.024
**PTPN5**	8.00	0.005	**DUSP2**	10.13	0.024	**SSH1**	1.45	0.033
**DUSP15**	8.51	0.001	**PTPRH**	10.27	0.007	**DUSP23**	1.50	0.009
**DUSP18**	9.86	0.025	**DUSP15**	13.10	0.001	**PTPRR**	1.53	0.068
**PTPRR**	14.62	0.009	**DUSP6**	15.69	0.010	**TNS3**	1.53	0.042
**DUSP9**	26.43	0.008	**DUSP9**	30.83	0.008	**PTPRN**	1.60	0.020
**PTPN22**	37.12	0.057	**PTPN22**	78.50	0.055	**PTPN5**	1.61	0.005
**PTPRQ2**	164.27	0.012	**PTPRQ2**	243.77	0.012	**DUSP19**	1.64	0.043
**DUSP19**	ND	ND	**DUSP19**	ND	ND	**PTPN21**	1.65	0.033
**MTMR8**	ND	ND	**MTMR8**	ND	ND	**PTPN23**	1.76	0.105
**MTMR11**	ND	ND	**MTMR11**	ND	ND	**DUSP18**	1.90	0.020
**PTPN6**	ND	ND	**PTPN6**	ND	ND	**PTPRT**	2.00	0.002

ND: Non Determined; WML: White Matter lesioned; CWM: Control White Matter; NAWM: Normal-appearing White Matter
