# Supplementary material for: Identification of VHY/Dusp15 as a Regulator of Oligodendrocyte Differentiation through a Systematic Genomics Approach
Source: PLoS One. 2012 Jul 11;7(7):e40457. doi: 10.1371/journal.pone.0040457 (PMC3394735; doi:10.1371/journal.pone.0040457)
Supplement: Table S3 — Differential gene expression of PTP family members in MS grey matter lesions. (PDF) [file pone.0040457.s003.pdf]

Table S3. Differential gene expression of PTP family members in MS grey matter lesions

	GML vs CGM			GML vs NAGM			NAGM vs CGM	
	**Fold**	**Pvalue**		**Fold**	**Pvalue**		**Fold**	**Pvalue**
**DUSP2**	-7.79	0.000	**CDC25C**	-5.25	0.007	**DUSP1**	-5.65	0.003
**CDC25C**	-4.66	0.317	**DUSP2**	-1.99	0.003	**DUSP5**	-4.94	0.005
**DUSP6**	-3.31	0.011	**MTMR7**	-1.69	0.006	**DUSP2**	-3.91	0.000
**CDKN3**	-2.80	0.078	**DUSP4**	-1.59	0.017	**DUSP6**	-3.36	0.010
**DUSP4**	-2.77	0.010	**DUSP12**	-1.56	0.009	**TENC1**	-2.25	0.009
**DUSP5**	-2.47	0.013	**ACP1**	-1.49	0.007	**PTPRD**	-2.21	0.022
**DUSP1**	-1.68	0.011	**CDKN3**	-1.43	0.270	**PTPN18**	-2.15	0.101
**PTP4A3**	-1.67	0.031	**MTMR4**	-1.42	0.006	**PTP4A3**	-2.04	0.003
**PTP4A1**	-1.42	0.011	**PTPRR**	-1.35	0.013	**DUSP18**	-1.98	0.018
**MTMR11**	-1.38	0.099	**PTP4A1**	-1.34	0.001	**CDKN3**	-1.96	0.025
**PTPN5**	-1.35	0.060	**PTPRN**	-1.34	0.010	**PTPN7**	-1.91	0.299
**PTP4A2**	-1.35	0.043	**PTPN1**	-1.31	0.139	**PTPN6**	-1.86	0.022
**PTPN3**	-1.34	0.051	**MTMR1**	-1.29	0.028	**CDC14b**	-1.85	0.092
**PTPN1**	-1.29	0.255	**PTPRO**	-1.28	0.009	**DUSP8**	-1.79	0.003
**DUSP12**	-1.29	0.130	**DUSP11**	-1.23	0.050	**PTPN3**	-1.79	0.034
**PTPRN**	-1.23	0.055	**PTPDC1**	-1.09	0.212	**PTPN22**	-1.76	0.044
**MTMR8**	-1.23	0.124	**PTPRH**	-1.07	0.419	**CDC14A**	-1.76	0.010
**PTPRN2**	-1.18	0.086	**PTPRQ2**	-1.06	0.094	**DUSP4**	-1.74	0.029
**MTMR7**	-1.16	0.102	**MTMR2**	-1.05	0.012	**DUSP15**	-1.72	0.042
**DUSP14**	-1.10	0.275	**PTPN4**	-1.05	0.190	**TNS1**	-1.70	0.015
**PTPN6**	-1.10	0.344	**DUSP10**	-1.03	0.443	**PTPN12**	-1.69	0.020
**MTMR9**	-1.07	0.342	**DUSP19**	-1.01	0.467	**PTPRS**	-1.67	0.004
**MTMR4**	-1.06	0.056	**DUSP3**	-1.00	0.494	**PTP4A2**	-1.64	0.024
**PTPRH**	-1.04	0.474	**DUSP22**	1.00	0.497	**SBF2**	-1.53	0.004
**CDC14b**	-1.03	0.460	**SSH2**	1.01	0.393	**PTPRZ1**	-1.52	0.041
**CDC25A**	-1.03	0.485	**DUSP6**	1.02	0.244	**MTMR11**	-1.51	0.032
**PTPN11**	-1.03	0.284	**MTMR6**	1.06	0.270	**hEPM2A**	-1.47	0.007
**PTPMT1**	-1.02	0.391	**DUSP14**	1.07	0.264	**PTPN5**	-1.46	0.038
**PTPDC1**	-1.01	0.467	**PTEN**	1.07	0.181	**TNS3**	-1.45	0.006
**DUSP15**	-1.00	0.495	**PTPN5**	1.08	0.066	**MTMR3**	-1.43	0.001
**DUSP3**	1.01	0.416	**MTMR8**	1.08	0.388	**PTPRG**	-1.43	0.054
**PTPRZ1**	1.03	0.212	**MTMR11**	1.09	0.314	**PTPN21**	-1.43	0.045
**hEPM2A**	1.03	0.362	**PTPRA**	1.11	0.040	**DUSP23**	-1.42	0.022
**MTMR1**	1.03	0.302	**PTPN11**	1.13	0.045	**DUSP9**	-1.37	0.216
**PTPRR**	1.04	0.367	**PTPMT1**	1.13	0.029	**PTPRM**	-1.37	0.016
**PTPRO**	1.04	0.389	**MTMR9**	1.14	0.040	**PTPRN2**	-1.37	0.033
**SBF2**	1.06	0.220	**PTPRN2**	1.15	0.061	**PTPN13**	-1.36	0.095
**PTPRG**	1.11	0.112	**hRNGTT**	1.19	0.050	**PTPN9**	-1.34	0.102
**PTPRM**	1.11	0.129	**CDC25A**	1.19	0.345	**MTMR8**	-1.33	0.181
**MTMR6**	1.12	0.077	**DUSP7**	1.20	0.022	**PTPRJ**	-1.31	0.028
**PTPRQ2**	1.13	0.247	**PTP4A2**	1.21	0.026	**PTPRT**	-1.27	0.062
**SSH3**	1.15	0.105	**PTPN2**	1.22	0.115	**STYXL1**	-1.24	0.036
**PTPN22**	1.15	0.373	**PTP4A3**	1.22	0.114	**CDC25A**	-1.23	0.344
**PTPRE**	1.15	0.069	**PTPN23**	1.25	0.109	**MTMR9**	-1.22	0.201
**PTEN**	1.16	0.028	**CDC25B**	1.25	0.031	**SSH3**	-1.19	0.030
**MTMR12**	1.18	0.027	**PTPRE**	1.27	0.045	**PTPRU**	-1.19	0.142
**DUSP10**	1.18	0.247	**PTPN14**	1.29	0.034	**DUSP14**	-1.18	0.160
**ACP1**	1.18	0.119	**MTMR12**	1.32	0.003	**PTPN11**	-1.16	0.033
**PTPRJ**	1.19	0.064	**PTPN3**	1.33	0.015	**PTPMT1**	-1.16	0.041
**PTPN23**	1.19	0.077	**DUSP16**	1.34	0.060	**SBF1**	-1.14	0.110

**PTPRA**	1.22	0.011	**SSH1**	1.37	0.048	**MTMR12**	-1.12	0.099
**DUSP22**	1.22	0.040	**SSH3**	1.37	0.008	**PTPRE**	-1.10	0.149
**hRNGTT**	1.23	0.091	**PTPRC**	1.38	0.109	**PTP4A1**	-1.05	0.162
**STYXL1**	1.23	0.027	**PTPRF**	1.40	0.001	**PTPN23**	-1.04	0.306
**DUSP11**	1.24	0.110	**SBF1**	1.45	0.015	**PTPRF**	-1.04	0.284
**SSH2**	1.25	0.074	**STYX**	1.46	0.007	**PTPRC**	-1.00	0.427
**PTPN13**	1.26	0.068	**hEPM2A**	1.51	0.007	**MTM1**	1.00	0.500
**PTPN18**	1.27	0.182	**PTPRM**	1.53	0.003	**PTPN1**	1.01	0.469
**SBF1**	1.27	0.008	**STYXL1**	1.53	0.012	**DUSP3**	1.01	0.358
**PTPN4**	1.28	0.079	**PTPRJ**	1.56	0.041	**PTPRK**	1.02	0.455
**PTPN9**	1.28	0.057	**PTPRZ1**	1.56	0.024	**PTPRH**	1.03	0.484
**PTPRD**	1.28	0.115	**PTPRK**	1.57	0.007	**hRNGTT**	1.04	0.405
**PTPRS**	1.29	0.041	**PTPRG**	1.59	0.006	**CDC25B**	1.04	0.315
**DUSP8**	1.29	0.008	**SBF2**	1.63	0.005	**MTMR6**	1.05	0.195
**CDC25B**	1.30	0.033	**PTPRT**	1.67	0.010	**STYX**	1.06	0.372
**DUSP7**	1.31	0.019	**MTM1**	1.67	0.064	**PTPDC1**	1.07	0.257
**PTPRT**	1.32	0.020	**PTPN6**	1.69	0.037	**PTEN**	1.08	0.188
**PTPRF**	1.35	0.015	**DUSP15**	1.71	0.044	**PTPRN**	1.09	0.216
**PTPRC**	1.37	0.100	**PTPN9**	1.72	0.002	**DUSP7**	1.09	0.207
**DUSP18**	1.38	0.063	**MTMR10**	1.72	0.005	**PTPRA**	1.10	0.054
**MTMR2**	1.38	0.032	**PTPN13**	1.72	0.023	**CDC25C**	1.13	0.431
**PTPN7**	1.47	0.287	**CDC14b**	1.79	0.034	**DUSP16**	1.15	0.074
**PTPN2**	1.52	0.009	**DUSP5**	2.00	0.023	**MTMR10**	1.15	0.014
**PTPN12**	1.52	0.010	**PTPN22**	2.03	0.089	**PTPRQ2**	1.20	0.138
**DUSP16**	1.53	0.021	**PTPRS**	2.15	0.003	**DUSP12**	1.21	0.107
**DUSP23**	1.54	0.007	**DUSP23**	2.19	0.001	**DUSP10**	1.22	0.013
**STYX**	1.55	0.030	**DUSP8**	2.32	0.003	**DUSP22**	1.22	0.125
**TENC1**	1.58	0.009	**DUSP9**	2.34	0.085	**PTPRB**	1.23	0.040
**PTPRK**	1.59	0.017	**MTMR3**	2.44	0.001	**SSH2**	1.23	0.104
**MTM1**	1.67	0.062	**TNS3**	2.45	0.002	**PTPN2**	1.25	0.055
**PTPN14**	1.68	0.046	**PTPN12**	2.58	0.003	**SSH1**	1.28	0.033
**TNS3**	1.69	0.002	**DUSP18**	2.72	0.004	**PTPN14**	1.31	0.062
**MTMR3**	1.70	0.002	**PTPN18**	2.72	0.010	**MTMR1**	1.33	0.016
**DUSP9**	1.70	0.189	**PTPN7**	2.82	ND	**MTMR4**	1.33	0.002
**TNS1**	1.74	0.007	**PTPRD**	2.83	0.004	**PTPRO**	1.34	0.031
**SSH1**	1.75	0.007	**PTPRU**	2.92	0.002	**PTPN4**	1.34	0.023
**DUSP19**	1.85	0.007	**TNS1**	2.96	0.000	**PTPRR**	1.40	0.006
**MTMR10**	1.98	0.002	**PTPN21**	3.33	0.054	**MTMR7**	1.45	0.015
**CDC14A**	2.26	0.009	**DUSP1**	3.36	0.001	**MTMR2**	1.46	0.027
**PTPN21**	2.33	0.058	**TENC1**	3.55	0.002	**DUSP11**	1.53	0.008
**PTPRU**	2.45	0.003	**CDC14A**	3.97	0.002	**ACP1**	1.77	0.011
**PTPRB**	10.06	0.169	**PTPRB**	8.19	0.175	**DUSP19**	1.88	0.016

ND: Non Determined; GML: Gray Matter lesioned; CGM: Control Gray Matter; NAGM: Normal-appearing Gray Matter
