# Supplementary material for: Identification of VHY/Dusp15 as a Regulator of Oligodendrocyte Differentiation through a Systematic Genomics Approach
Source: PLoS One. 2012 Jul 11;7(7):e40457. doi: 10.1371/journal.pone.0040457 (PMC3394735; doi:10.1371/journal.pone.0040457)
Supplement: Table S4 — PTP gene expression in spinal cord and cerebellum from MOG-induced EAE mice. Differential gene expression of PTP family members in EAE spinal cord and cerebellum lesions. (PDF) [file pone.0040457.s004.pdf]

Table S4. *PTP genes expression in spinal cord and cerebellum from MOG-induced EAE mice.* Differential gene expression of PTP family members in EAE spinal cord and cerebellum lesions

Mouse spinal cord

	EAE *vs* SHAM (D14)			EAE *vs* SHAM (D17)			EAE *s* SHAM (D28)	
	Fold	Pvalue		Fold	Pvalue		Fold	Pvalue
**Ptpn20**	-1.59	0.055	**Ptprd**	-2.44	0.001	**Ptprd**	-2.15	0.003
**Ptprh**	-1.47	0.081	**Dusp15**	-2.40	0.001	**Ptprr**	-2.13	0.012
**Mtmr2**	-1.37	0.030	**Ptprn2**	-2.24	0.000	**Epm2a**	-1.93	0.000
**Styxl1**	-1.35	0.214	**Ptprr**	-1.94	0.006	**Ptpre**	-1.89	0.008
**Ptpn4**	-1.27	0.181	**Ptpn13**	-1.89	0.023	**Ptpn13**	-1.82	0.003
**Ptprr**	-1.26	0.168	**Dusp8**	-1.75	0.006	**Dusp8**	-1.79	0.001
**Ptprd**	-1.25	0.260	**Ptpn3**	-1.72	0.003	**Mtmr7**	-1.79	0.006
**Ptpre**	-1.20	0.232	**Ptpn5**	-1.72	0.005	**Ptprn2**	-1.75	0.003
**Tns1**	-1.14	0.293	**Epm2a**	-1.72	0.004	**Dusp15**	-1.75	0.008
**Ptp4a1**	-1.13	0.101	**Ptprn**	-1.69	0.010	**Ptprm**	-1.74	0.020
**Dusp15**	-1.09	0.422	**Ptpn14**	-1.69	0.094	**Ptpn3**	-1.66	0.038
**Ptpmt1**	-1.05	0.164	**Mtmr7**	-1.63	0.011	**Dusp26**	-1.66	0.009
**Dusp8**	-1.04	0.455	**Ptprm**	-1.59	0.006	**Mtmr2**	-1.59	0.015
**Epm2a**	-1.04	0.129	**Ptprs**	-1.56	0.008	**Cdc14b**	-1.58	0.002
**Mtmr7**	-1.03	0.208	**Ptpre**	-1.54	0.013	**Ptpn5**	-1.57	0.013
**Ptpdc1**	-1.03	0.192	**Tenc1**	-1.52	0.012	**Dusp4**	-1.56	0.038
**Sbf2**	-1.00	0.452	**Ptpdc1**	-1.47	0.008	**Dusp10**	-1.52	0.011
**Ptprz1**	1.00	0.356	**Ptprh**	-1.47	0.018	**Tenc1**	-1.46	0.010
**Ptprm**	1.00	0.471	**Ptprz1**	-1.45	0.022	**Ptpdc1**	-1.45	0.001
**Ptprn**	1.00	0.486	**Tns1**	-1.43	0.056	**Ptprs**	-1.42	0.006
**Ptprf**	1.03	0.314	**Mtmr9**	-1.40	0.026	**Ptprz1**	-1.41	0.002
**Dusp1**	1.04	0.215	**Mtmr2**	-1.39	0.003	**Ptp4a1**	-1.40	0.088
**Dusp12**	1.05	0.122	**Sbf1**	-1.31	0.038	**Mtmr6**	-1.39	0.050
**Dusp10**	1.05	0.349	**Ptpn20**	-1.31	0.302	**Mtmr4**	-1.38	0.010
**Mtm1**	1.05	0.311	**Cdc14b**	-1.28	0.001	**Mtmr10**	-1.35	0.028
**Dusp19**	1.05	0.316	**Ptprt**	-1.27	0.056	**Styxl1**	-1.34	0.163
**Ptpn3**	1.06	0.370	**Ptpro**	-1.27	0.064	**Dusp1**	-1.33	0.049
**Mtmr10**	1.07	0.021	**Dusp10**	-1.25	0.186	**Dusp22**	-1.31	0.081
**Dusp23**	1.08	0.388	**Ptprg**	-1.23	0.133	**Ptprb**	-1.31	0.029
**Ptprg**	1.10	0.318	**Ptprb**	-1.21	0.065	**Ptprg**	-1.30	0.010
**Ptprn2**	1.10	0.312	**Ptprf**	-1.21	0.104	**Mtmr9**	-1.29	0.018
**Dusp22**	1.10	0.150	**Dusp4**	-1.20	0.182	**Ptprn**	-1.27	0.001
**Acp1**	1.12	0.269	**Ptp4a1**	-1.20	0.118	**Sbf1**	-1.25	0.087
**Dusp14**	1.12	0.347	**Dusp26**	-1.19	0.147	**Ptprt**	-1.24	0.039
**Ptprt**	1.12	0.278	**Mtmr4**	-1.19	0.158	**Ptpra**	-1.24	0.042
**Dusp26**	1.13	0.095	**Ptprk**	-1.15	0.153	**Ptprk**	-1.23	0.126
**Ptpn5**	1.13	0.295	**Dusp12**	-1.14	0.128	**Mtmr3**	-1.22	0.123
**Pten**	1.14	0.167	**Dusp14**	-1.13	0.144	**Ptpro**	-1.22	0.218
**Ptp4a3**	1.14	0.168	**Mtmr6**	-1.13	0.215	**Tns1**	-1.17	0.244
**Dusp18**	1.15	0.129	**Dusp7**	-1.06	0.231	**Ssh1**	-1.16	0.121
**Ptpra**	1.15	0.055	**Dusp22**	-1.05	0.321	**Dusp19**	-1.14	0.336
**Ptprj**	1.16	0.195	**Mtmr10**	-1.01	0.481	**Rngtt**	-1.13	0.097
**Sbf1**	1.20	0.226	**Ptpra**	-1.00	0.493	**Dusp12**	-1.12	0.156
**Dusp4**	1.21	0.093	**Rngtt**	1.04	0.316	**Ptp4a3**	-1.11	0.068

**Cdc14b**	1.22	0.015
**Ptprs**	1.22	0.155
**Tenc1**	1.22	0.027
**Dusp7**	1.23	0.005
**Ssh1**	1.23	0.198
**Cdc25b**	1.23	0.288
**Ptpn13**	1.24	0.321
**Mtmr4**	1.24	0.078
**Mtmr1**	1.24	0.019
**Ptprb**	1.25	0.051
**Rngtt**	1.28	0.010
**Mtmr6**	1.28	0.011
**Mtmr12**	1.28	0.006
**Dusp11**	1.29	0.054
**Ptpn9**	1.29	0.012
**Dusp3**	1.34	0.009
**Ptpn23**	1.35	0.077
**Mtmr9**	1.36	0.082
**Ptpn21**	1.38	0.065
**Ptpn11**	1.38	0.063
**Styx**	1.42	0.106
**Ptpro**	1.45	0.028
**Ptp4a2**	1.46	0.086
**Mtmr3**	1.47	0.123
**Dusp16**	1.48	0.022
**Ptpn14**	1.52	0.015
**Ssh2**	1.63	0.084
**Dusp2**	1.63	0.115
**Ptprk**	1.63	0.009
**Cdc25a**	1.69	0.008
**Ptpn2**	1.72	0.124
**Cdc14a**	1.72	0.125
**Ssh3**	1.74	0.089
**Dusp6**	1.79	0.103
**Ptpn12**	1.81	0.078
**Mtmr11**	2.30	0.065
**Ptpn22**	2.73	0.135
**Tns3**	2.79	0.048
**Dusp5**	3.50	0.118
**Ptprv**	4.20	0.083
**Cdkn3**	4.80	0.076
**Ptpn1**	4.93	0.076
**Cdc25c**	5.77	0.107
**Ptprc**	7.44	0.079
**Ptpn6**	10.74	0.088
**Ptpn18**	ND	ND
**Ptpn7**	ND	ND
**Ptprq**	ND	ND

**Mtmr3**	1.04	0.415
**Ptpn4**	1.07	0.189
**Ptpmt1**	1.08	0.153
**Ptpn11**	1.08	0.303
**Ssh1**	1.08	0.364
**Dusp18**	1.09	0.369
**Sbf2**	1.09	0.168
**Dusp16**	1.12	0.307
**Acp1**	1.19	0.140
**Cdc25a**	1.19	0.102
**Dusp3**	1.19	0.187
**Ptpn23**	1.23	0.079
**Dusp1**	1.24	0.241
**Ptpn9**	1.27	0.092
**Mtmr11**	1.31	0.217
**Ptp4a3**	1.31	0.013
**Dusp11**	1.32	0.006
**Pten**	1.38	0.043
**Mtmr12**	1.38	0.026
**Mtmr1**	1.40	0.025
**Dusp23**	1.41	0.018
**Ssh3**	1.45	0.069
**Ptp4a2**	1.47	0.005
**Ptpn21**	1.47	0.000
**Styx**	1.49	0.009
**Mtm1**	1.55	0.031
**Styxl1**	1.56	0.102
**Cdc14a**	1.57	0.021
**Ptpn2**	1.74	0.014
**Ssh2**	1.76	0.015
**Cdc25b**	2.03	0.015
**Ptprj**	2.14	0.001
**Ptpn12**	2.20	0.011
**Dusp6**	2.42	0.003
**Dusp19**	2.75	0.015
**Dusp5**	3.22	0.004
**Tns3**	3.37	0.000
**Cdkn3**	4.85	0.014
**Cdc25c**	5.52	0.026
**Dusp2**	6.03	0.005
**Ptpn1**	6.20	0.000
**Ptpn22**	10.69	0.001
**Ptprc**	17.96	0.000
**Ptpn6**	42.75	0.000
**Ptpn18**	ND	ND
**Ptpn7**	ND	ND
**Ptprq**	ND	ND
**Ptprv**	ND	ND

**Sbf2**	-1.11	0.054
**Ptpn21**	-1.09	0.369
**Cdc25b**	-1.09	0.089
**Dusp14**	-1.09	0.082
**Dusp3**	-1.08	0.257
**Ptpn4**	-1.07	0.264
**Dusp7**	-1.05	0.242
**Ptpn11**	-1.05	0.260
**Mtmr1**	-1.04	0.366
**Ptpn9**	-1.03	0.428
**Pten**	1.01	0.473
**Ptprh**	1.01	0.475
**Ptpmt1**	1.03	0.331
**Dusp16**	1.03	0.352
**Ptpn14**	1.04	0.442
**Cdc25a**	1.06	0.293
**Ptpn23**	1.11	0.212
**Acp1**	1.13	0.250
**Dusp23**	1.16	0.166
**Mtmr12**	1.16	0.151
**Mtm1**	1.19	0.226
**Ssh2**	1.22	0.083
**Mtmr11**	1.22	0.170
**Dusp11**	1.23	0.126
**Ptprf**	1.24	0.030
**Styx**	1.27	0.034
**Ptp4a2**	1.39	0.017
**Ssh3**	1.42	0.020
**Ptpn12**	1.49	0.052
**Ptprj**	1.54	0.039
**Ptpn20**	1.68	0.071
**Dusp18**	1.68	0.019
**Cdc14a**	1.73	0.044
**Dusp6**	1.74	0.052
**Ptpn2**	1.84	0.049
**Ptpn18**	2.02	0.075
**Ptprq**	2.36	0.076
**Tns3**	2.85	0.003
**Dusp5**	3.16	0.033
**Dusp2**	3.27	0.004
**Cdc25c**	3.82	0.074
**Cdkn3**	3.99	0.029
**Ptpn1**	4.09	0.003
**Ptpn22**	6.73	0.005
**Ptprc**	12.76	0.006
**Ptpn7**	13.42	0.013
**Ptpn6**	22.60	0.002
**Ptprv**	ND	ND

ND: Non Determined

Mouse cerebellum								
	EAE *vs* SHAM (D14)			EAE *vs* SHAM (D17)			EAE *s* SHAM (D28)	
	**Fold**	**Pvalue**		**Fold**	**Pvalue**		**Fold**	**Pvalue**
**Dusp19**	-4.22	0.008	**Dusp5**	-2.06	0.009	**Ptpn4**	-1.84	0.184
**Ptpn18**	-2.23	0.017	**Ptprd**	-1.88	0.010	**Ptprv**	-1.83	0.086
**Ptpn4**	-2.10	0.171	**Ptprr**	-1.87	0.033	**Dusp19**	-1.50	0.103
**Dusp4**	-1.73	0.054	**Ptpn5**	-1.86	0.001	**Ptpn14**	-1.35	0.133
**Dusp5**	-1.73	0.076	**Ptpro**	-1.85	0.023	**Dusp1**	-1.25	0.155
**Cdc25b**	-1.68	0.063	**Ptprk**	-1.84	0.010	**Cdkn3**	-1.19	0.247
**Dusp1**	-1.63	0.004	**Ptpn13**	-1.83	0.031	**Ptprf**	-1.15	0.165
**Mtmr3**	-1.58	0.125	**Mtmr2**	-1.79	0.003	**Ptpn18**	-1.13	0.278
**Dusp2**	-1.47	0.288	**Ptprn2**	-1.78	0.004	**Dusp4**	-1.11	0.351
**Ptpn23**	-1.39	0.176	**Ptprm**	-1.68	0.001	**Dusp18**	-1.05	0.403
**Ssh3**	-1.38	0.002	**Mtmr7**	-1.67	0.007	**Cdc14b**	-1.05	0.386
**Ptp4a3**	-1.37	0.179	**Ptprs**	-1.65	0.005	**Dusp26**	-1.04	0.412
**Acp1**	-1.36	0.328	**Rngtt**	-1.64	0.004	**Dusp12**	-1.02	0.446
**Dusp8**	-1.36	0.116	**Dusp10**	-1.63	0.003	**Mtmr3**	-1.02	0.462
**Mtmr1**	-1.35	0.113	**Dusp15**	-1.58	0.007	**Ssh3**	-1.01	0.374
**Ptprb**	-1.35	0.366	**Mtmr11**	-1.54	0.031	**Tenc1**	-1.00	0.481
**Ptprn2**	-1.35	0.267	**Sbf1**	-1.52	0.036	**Cdc25b**	1.01	0.484
**Ptprj**	-1.35	0.095	**Dusp8**	-1.51	0.001	**Dusp5**	1.02	0.449
**Ptprd**	-1.32	0.095	**Mtmr9**	-1.51	0.004	**Ptpn9**	1.02	0.411
**Ptprr**	-1.31	0.155	**Dusp1**	-1.48	0.012	**Tns3**	1.03	0.447
**Mtmr4**	-1.29	0.120	**Mtmr4**	-1.48	0.002	**Ptp4a1**	1.03	0.399
**Ptprm**	-1.29	0.258	**Mtmr6**	-1.47	0.003	**Ptp4a3**	1.03	0.364
**Ptpn9**	-1.28	0.041	**Ptprn**	-1.46	0.017	**Tns1**	1.04	0.292
**Ptpn22**	-1.28	0.239	**Epm2a**	-1.44	0.071	**Dusp22**	1.05	0.342
**Mtmr12**	-1.25	0.153	**Dusp4**	-1.42	0.080	**Dusp3**	1.06	0.056
**Ptpra**	-1.24	0.264	**Dusp18**	-1.42	0.071	**Dusp8**	1.06	0.322
**Mtmr10**	-1.24	0.058	**Ssh1**	-1.41	0.020	**Ptpn22**	1.07	0.287
**Dusp12**	-1.24	0.131	**Tenc1**	-1.40	0.063	**Dusp14**	1.07	0.204
**Sbf2**	-1.24	0.289	**Ptpn14**	-1.38	0.111	**Ptpra**	1.07	0.156
**Cdc14b**	-1.22	0.243	**Ptpn9**	-1.37	0.031	**Ssh1**	1.07	0.207
**Ptpn14**	-1.19	0.498	**Mtmr12**	-1.36	0.078	**Ptpmt1**	1.07	0.243
**Ptprz1**	-1.19	0.328	**Ptprb**	-1.35	0.037	**Mtmr4**	1.08	0.281
**Sbf1**	-1.19	0.399	**Ptpdc1**	-1.29	0.020	**Ptprn2**	1.08	0.350
**Dusp3**	-1.18	0.452	**Ptpra**	-1.28	0.007	**Dusp10**	1.09	0.215
**Epm2a**	-1.18	0.376	**Pten**	-1.26	0.001	**Ptprd**	1.09	0.126
**Pten**	-1.17	0.219	**Dusp7**	-1.26	0.076	**Ptpn23**	1.09	0.233
**Ptprn**	-1.17	0.415	**Dusp11**	-1.25	0.018	**Ptpn5**	1.09	0.346
**Mtmr7**	-1.16	0.410	**Dusp16**	-1.24	0.009	**Mtmr6**	1.09	0.291
**Dusp10**	-1.15	0.479	**Dusp12**	-1.24	0.002	**Ptprr**	1.09	0.198
**Ptpmt1**	-1.14	0.061	**Ptpn23**	-1.21	0.166	**Dusp15**	1.10	0.187
**Mtmr2**	-1.12	0.016	**Ptprz1**	-1.20	0.058	**Dusp11**	1.10	0.175
**Ptpn11**	-1.12	0.467	**Ptprg**	-1.20	0.009	**Dusp16**	1.10	0.199
**Ptprs**	-1.12	0.478	**Mtmr3**	-1.19	0.088	**Ptprz1**	1.10	0.106
**Ptpn3**	-1.12	0.411	**Dusp26**	-1.19	0.185	**Ptpn11**	1.10	0.173
**Ssh1**	-1.11	0.429	**Sbf2**	-1.18	0.006	**Mtmr1**	1.11	0.148
**Dusp11**	-1.11	0.432	**Ssh2**	-1.17	0.068	**Ptprb**	1.11	0.183
**Dusp16**	-1.11	0.430	**Mtmr10**	-1.15	0.138	**Ssh2**	1.12	0.270

**Ptprg**	-1.10	0.464	**Dusp6**	-1.14	0.158	**Ptprc**	1.12	0.343
**Rngtt**	-1.10	0.409	**Ptp4a1**	-1.14	0.098	**Sbf1**	1.12	0.228
**Dusp15**	-1.07	0.374	**Dusp3**	-1.13	0.209	**Ptpro**	1.12	0.323
**Ptprt**	-1.06	0.300	**Dusp22**	-1.13	0.097	**Mtmr2**	1.12	0.037
**Mtmr6**	-1.04	0.434	**Cdc25a**	-1.12	0.209	**Mtmr12**	1.13	0.126
**Dusp22**	-1.03	0.338	**Ptpre**	-1.10	0.326	**Ptpn1**	1.13	0.237
**Ptpn7**	ND	ND	**Ptpn21**	-1.09	0.310	**Epm2a**	1.13	0.300
**Ptpre**	1.01	0.261	**Mtmr1**	-1.08	0.277	**Ptpn2**	1.13	0.191
**Ptpn21**	1.01	0.304	**Ptp4a2**	-1.07	0.006	**Ptprs**	1.13	0.174
**Dusp26**	1.01	0.351	**Ptpn12**	-1.07	0.318	**Ptprn**	1.13	0.205
**Ssh2**	1.01	0.277	**Ptpn3**	-1.05	0.417	**Pten**	1.14	0.044
**Ptpn12**	1.01	0.299	**Ptprt**	-1.05	0.349	**Ptprt**	1.14	0.243
**Mtmr9**	1.02	0.217	**Ptpn2**	-1.03	0.436	**Ptpn12**	1.14	0.057
**Ptpn13**	1.03	0.443	**Acp1**	-1.03	0.310	**Mtmr7**	1.16	0.019
**Ptpn2**	1.07	0.371	**Ptprq**	-1.01	0.479	**Ptpre**	1.17	0.159
**Ptpn5**	1.07	0.321	**Ptpmt1**	-1.00	0.488	**Acp1**	1.17	0.010
**Ptp4a1**	1.08	0.326	**Ptprj**	1.00	0.500	**Ptp4a2**	1.17	0.063
**Dusp23**	1.08	0.213	**Ssh3**	1.01	0.474	**Rngtt**	1.17	0.076
**Tns1**	1.11	0.279	**Ptp4a3**	1.01	0.443	**Sbf2**	1.18	0.015
**Dusp18**	1.13	0.160	**Ptpn11**	1.02	0.400	**Ptprk**	1.19	0.326
**Dusp7**	1.13	0.184	**Styx**	1.05	0.403	**Mtm1**	1.21	0.046
**Cdc25a**	1.13	0.222	**Cdc14b**	1.06	0.280	**Ptprm**	1.21	0.034
**Ptp4a2**	1.17	0.003	**Ptpn22**	1.06	0.368	**Mtmr10**	1.23	0.008
**Dusp14**	1.20	0.275	**Ptprv**	1.08	0.426	**Cdc25a**	1.23	0.162
**Mtm1**	1.21	0.031	**Dusp14**	1.11	0.063	**Dusp6**	1.25	0.090
**Mtmr11**	1.22	0.297	**Dusp23**	1.14	0.146	**Ptprh**	1.26	0.148
**Ptprk**	1.23	0.169	**Tns1**	1.16	0.023	**Ptprg**	1.26	0.116
**Dusp6**	1.24	0.159	**Ptprf**	1.20	0.006	**Ptprj**	1.27	0.029
**Ptpdc1**	1.28	0.211	**Ptprh**	1.23	0.296	**Dusp7**	1.27	0.030
**Styx**	1.29	0.215	**Mtm1**	1.28	0.004	**Dusp23**	1.28	0.014
**Ptprf**	1.32	0.062	**Cdc25c**	1.33	0.075	**Ptpn3**	1.28	0.090
**Ptprv**	1.34	0.151	**Cdc14a**	1.34	0.071	**Ptpn6**	1.28	0.098
**Tenc1**	1.42	0.136	**Tns3**	1.40	0.010	**Styx**	1.29	0.106
**Ptprh**	1.49	0.258	**Ptpn4**	1.45	0.038	**Mtmr9**	1.29	0.074
**Ptpro**	1.52	0.126	**Ptpn1**	1.62	0.032	**Ptpn21**	1.30	0.003
**Ptpn20**	1.53	0.288	**Styxl1**	2.07	0.145	**Mtmr11**	1.36	0.102
**Cdc14a**	1.60	0.149	**Ptpn18**	2.36	0.161	**Cdc14a**	1.38	0.033
**Cdc25c**	1.77	0.042	**Ptpn20**	2.53	0.052	**Ptpn13**	1.40	0.113
**Ptpn1**	1.78	0.151	**Cdkn3**	3.08	0.020	**Styxl1**	1.40	0.012
**Tns3**	1.87	0.118	**Ptpn7**	3.18	0.054	**Ptpn7**	1.42	0.279
**Ptprq**	2.18	0.276	**Cdc25b**	3.63	0.000	**Ptpdc1**	1.50	0.024
**Styxl1**	2.24	0.196	**Ptprc**	3.78	0.010	**Ptprq**	2.14	0.307
**Cdkn3**	2.48	0.07	**Ptpn6**	7.27	0.01	**Cdc25c**	2.69	0.24
**Ptprc**	4.17	0.035	**Dusp2**	18.72	0.053	**Dusp2**	3.28	0.070
**Ptpn6**	5.42	0.046	**Dusp19**	30.01	0.002	**Ptpn20**	5.12	0.092

ND: Non Determined
