# Supplementary material for: Identification of VHY/Dusp15 as a Regulator of Oligodendrocyte Differentiation through a Systematic Genomics Approach
Source: PLoS One. 2012 Jul 11;7(7):e40457. doi: 10.1371/journal.pone.0040457 (PMC3394735; doi:10.1371/journal.pone.0040457)
Supplement: Table S5 — PTP gene expression during OL differentiation in mouse mixed cortical cultures. Differential gene expression of PTP family members in mixed cortical culture undergoing OL differentiation. (PDF) [file pone.0040457.s005.pdf]

Table S5. *PTP gene expression during OL differentiation in mouse mixed cortical cultures*

Differential gene expression of PTP family members in mixed cortical culture undergoing OL differentiation

	DIV8/DIV5			DIV12/DIV5			DIV15/DIV5	
	**Fold**	**Pvalue**		**Fold**	**Pvalue**		**Fold**	**Pvalue**
**Mtmr12**	-4.99	0.036	**Mtmr11**	-7.41	0.054	**Dusp2**	-8.45	0.002
**Cdc25c**	-3.75	0.070	**Dusp4**	-4.64	0.016	**Ptpro**	-4.27	0.013
**Ptpn23**	-3.33	0.022	**Dusp2**	-4.18	0.004	**Ssh1**	-2.71	0.064
**Ptpn14**	-2.11	0.026	**Cdc25c**	-4.09	0.032	**Dusp8**	-2.50	0.018
**Ptpn6**	-2.00	ND	**Ptpn6**	-3.36	ND	**Cdc25c**	-2.27	0.022
**Dusp4**	-1.95	0.104	**Ptpn5**	-3.29	0.010	**Ptp4a1**	-2.11	0.016
**Dusp1**	-1.75	0.018	**Ptprz1**	-3.00	0.015	**Ptpn6**	-1.95	ND
**Ptprk**	-1.57	0.045	**Ptpro**	-2.85	0.008	**Styx**	-1.84	0.000
**Ssh1**	-1.40	0.188	**Ptpn13**	-2.59	0.018	**Ssh2**	-1.81	0.034
**Ptpro**	-1.28	0.147	**Mtmr12**	-2.53	0.093	**Cdc25a**	-1.73	0.059
**Tns1**	-1.25	0.206	**Tenc1**	-2.49	0.028	**Ptpn23**	-1.71	0.045
**Ptprm**	-1.19	0.222	**Ptprd**	-2.43	0.013	**Dusp12**	-1.71	0.060
**Ptpn5**	-1.12	0.306	**Ptpn9**	-2.26	0.040	**Rngtt**	-1.71	0.044
**Dusp8**	-1.11	0.374	**Dusp8**	-2.26	0.016	**Ptprh**	-1.69	0.108
**Dusp12**	-1.11	0.228	**Mtmr6**	-2.24	0.023	**Sbf2**	-1.54	0.075
**Mtmr2**	-1.09	0.278	**Ptpn12**	-2.11	0.024	**Dusp1**	-1.50	0.003
**Cdc25a**	-1.09	0.303	**Dusp18**	-2.07	0.011	**Ptpn5**	-1.46	0.019
**Sbf2**	-1.08	0.225	**Rngtt**	-2.00	0.049	**Ptprd**	-1.46	0.067
**Ptprz1**	-1.07	0.312	**Mtmr2**	-1.93	0.059	**Mtmr4**	-1.40	0.161
**Ptpn4**	-1.07	0.330	**Dusp12**	-1.90	0.040	**Ptpn9**	-1.38	0.137
**Ssh2**	-1.05	0.439	**Ptpn22**	-1.81	0.025	**Ptpn21**	-1.31	0.112
**Ptpn9**	-1.03	0.447	**Ssh1**	-1.79	0.134	**Dusp22**	-1.30	0.220
**Ptpn12**	-1.02	0.433	**Cdc25a**	-1.65	0.071	**Cdkn3**	-1.29	0.014
**Cdc25b**	1.02	0.307	**Dusp6**	-1.62	0.042	**Ptpn12**	-1.26	0.175
**Dusp16**	1.02	0.436	**Mtmr4**	-1.59	0.084	**Dusp4**	-1.26	0.164
**Mtmr1**	1.03	0.250	**Ssh2**	-1.56	0.012	**Cdc25b**	-1.24	0.162
**Ptpn1**	1.05	0.193	**Sbf2**	-1.55	0.080	**Ptprs**	-1.23	0.114
**Dusp2**	1.06	0.314	**Ptpn1**	-1.43	0.013	**Mtmr9**	-1.21	0.192
**Rngtt**	1.08	0.433	**Ptpn11**	-1.31	0.120	**Dusp11**	-1.20	0.192
**Dusp18**	1.09	0.251	**Acp1**	-1.31	0.080	**Ptprz1**	-1.16	0.127
**Ptp4a2**	1.11	0.192	**Ptprk**	-1.31	0.156	**Ptprj**	-1.11	0.255
**Dusp22**	1.11	0.310	**Ptp4a1**	-1.27	0.110	**Mtmr6**	-1.06	0.187
**Mtmr6**	1.12	0.330	**Styx**	-1.26	0.016	**Ptpn1**	1.05	0.407
**Ptprh**	1.16	0.104	**Ptprh**	-1.24	0.405	**Ptp4a2**	1.05	0.295
**Cdc14b**	1.17	0.323	**Ptprm**	-1.23	0.084	**Acp1**	1.07	0.218
**Dusp10**	1.19	0.274	**Dusp11**	-1.22	0.021	**Dusp18**	1.09	0.427
**Ptpmt1**	1.19	0.079	**Cdkn3**	-1.19	0.230	**Mtmr3**	1.09	0.071
**Acp1**	1.20	0.283	**Ptpn23**	-1.15	0.247	**Ptpra**	1.10	0.233
**Ptprs**	1.23	0.244	**Ptp4a2**	-1.06	0.319	**Ptpn4**	1.10	0.148
**Ptpra**	1.24	0.198	**Dusp22**	-1.05	0.450	**Cdc14a**	1.11	0.339
**Ptprr**	1.27	0.149	**Mtmr9**	-1.05	0.398	**Ptpdc1**	1.12	0.126
**Dusp11**	1.28	0.030	**Ptpn21**	-1.05	0.365	**Pten**	1.19	0.106
**Mtmr9**	1.29	0.183	**Cdc14a**	-1.01	0.482	**Dusp10**	1.19	0.214
**Styx**	1.29	0.077	**Ptpra**	-1.00	0.496	**Ptpre**	1.20	0.294
**Ptprj**	1.29	0.160	**Ptprs**	1.03	0.498	**Ptprg**	1.23	0.159
**Ptprd**	1.30	0.238	**Mtm1**	1.04	0.436	**Dusp7**	1.25	0.188
**Mtmr4**	1.33	0.007	**Ptpn20**	1.05	0.404	**Dusp26**	1.26	0.018
**Ptpn21**	1.35	0.104	**Mtmr3**	1.06	0.417	**Ptprf**	1.26	0.257
**Ptpn11**	1.37	0.194	**Ptprj**	1.07	0.375	**Ptprm**	1.27	0.169

**Ptpn20**	1.47	0.142	**Dusp1**	1.09	0.137	**Mtmr12**	1.36	0.010
**Mtmr3**	1.47	0.040	**Pten**	1.20	0.105	**Ptpn11**	1.39	0.076
**Dusp7**	1.48	0.094	**Ptp4a3**	1.22	0.307	**Ptprn2**	1.52	0.160
**Mtmr11**	1.49	0.053	**Tns1**	1.22	0.073	**Ptp4a3**	1.59	0.131
**Ptprg**	1.52	0.088	**Ptprg**	1.23	0.258	**Ptprk**	1.65	0.346
**Dusp6**	1.52	0.031	**Dusp7**	1.24	0.148	**Ptpmt1**	1.66	0.002
**Ptpn18**	1.55	0.011	**Ptpn4**	1.28	0.148	**Tenc1**	1.66	0.147
**Dusp23**	1.55	0.117	**Ptprf**	1.28	0.200	**Mtmr2**	1.66	0.175
**Pten**	1.62	0.019	**Mtmr1**	1.31	0.072	**Mtmr1**	1.77	0.041
**Dusp3**	1.63	0.091	**Dusp16**	1.34	0.047	**Sbf1**	1.88	0.002
**Ptpn22**	1.67	0.003	**Sbf1**	1.35	0.123	**Dusp6**	2.08	0.195
**Cdkn3**	1.69	0.076	**Dusp10**	1.40	0.139	**Dusp16**	2.10	0.017
**Ptp4a3**	1.69	0.028	**Ptpdc1**	1.42	0.132	**Ptpn14**	2.33	0.007
**Ptp4a1**	1.77	0.042	**Ptprt**	1.42	0.010	**Ptprt**	2.50	0.007
**Cdc14a**	1.77	0.023	**Ptpn18**	1.50	0.114	**Dusp23**	2.52	0.055
**Ptprn2**	1.84	0.167	**Ptprr**	1.52	0.089	**Mtmr7**	2.55	0.020
**Ptpre**	1.89	0.023	**Ptpmt1**	1.59	0.000	**Mtmr11**	2.85	0.004
**Sbf1**	1.97	0.066	**Ptpn2**	1.89	0.193	**Ptprr**	2.91	0.036
**Dusp26**	1.97	0.040	**Dusp26**	2.07	0.016	**Ssh3**	3.02	0.010
**Ptpn2**	1.99	0.041	**Cdc25b**	2.09	0.039	**Ptpn13**	3.12	0.004
**Ptprt**	2.22	0.020	**Ptpn14**	2.14	0.068	**Ptpn2**	3.32	0.138
**Tenc1**	2.38	0.084	**Dusp3**	2.17	0.017	**Dusp3**	3.37	0.003
**Ptpdc1**	2.39	0.022	**Mtmr7**	2.38	0.022	**Tns1**	3.54	0.004
**Ptprf**	2.59	0.074	**Ptpre**	2.44	0.009	**Dusp14**	3.58	0.001
**Ptpn13**	2.75	0.027	**Ptprn2**	2.46	0.043	**Ptpn22**	3.67	0.213
**Dusp14**	2.83	0.065	**Dusp23**	2.71	0.003	**Ptpn18**	3.95	0.081
**Mtmr7**	3.05	0.002	**Dusp14**	2.98	0.026	**Tns3**	4.13	0.055
**Tns3**	3.36	0.019	**Cdc14b**	3.99	0.025	**Cdc14b**	4.69	0.026
**Ptprn**	3.68	0.026	**Ptprn**	4.01	0.027	**Epm2a**	5.52	0.012
**Ssh3**	3.82	0.002	**Ssh3**	4.40	0.015	**Mtm1**	5.60	0.126
**Epm2a**	3.86	0.044	**Tns3**	4.60	0.006	**Ptprn**	6.93	0.026
**Mtm1**	3.89	0.007	**Dusp5**	4.65	0.094	**Dusp15**	7.71	0.076
**Dusp15**	4.52	0.007	**Dusp15**	5.41	0.015	**Dusp5**	11.28	0.033
**Dusp5**	9.90	0.083	**Epm2a**	10.01	0.015	**Dusp19**	ND	ND
**Dusp19**	ND	ND	**Dusp19**	ND	ND	**Mtmr10**	ND	ND
**Mtmr10**	ND	ND	**Mtmr10**	ND	ND	**Ptpn20**	ND	ND
**Ptpn3**	ND	ND	**Ptpn3**	ND	ND	**Ptpn3**	ND	ND
**Ptpn7**	ND	ND	**Ptpn7**	ND	ND	**Ptpn7**	ND	ND
**Ptprb**	ND	ND	**Ptprb**	ND	ND	**Ptprb**	ND	ND
**Ptprc**	ND	ND	**Ptprc**	ND	ND	**Ptprc**	ND	ND
**Ptprq**	ND	ND	**Ptprq**	ND	ND	**Ptprq**	ND	ND
**Ptprv**	ND	ND	**Ptprv**	ND	ND	**Ptprv**	ND	ND
**Styxl1**	ND	ND	**Styxl1**	ND	ND	**Styxl1**	ND	ND

ND: Non Determined
